# Two‐Dimensional Noble‐Metal Chalcogenides and Phosphochalcogenides†

**DOI:** 10.1002/anie.201914886

**Published:** 2020-04-01

**Authors:** Roman Kempt, Agnieszka Kuc, Thomas Heine

**Affiliations:** ^1^ Faculty of Chemistry and Food Chemistry Technische Universität Dresden Bergstrasse 66 01069 Dresden Germany; ^2^ Institute of Resource Ecology Helmholtz-Zentrum Dresden-Rossendorf Permoserstrasse 15 04318 Leipzig Germany

**Keywords:** density functional calculations, noble-metal dichalcogenides, Raman spectroscopy, sensors, two-dimensional materials

## Abstract

Noble‐metal chalcogenides, dichalcogenides, and phosphochalcogenides are an emerging class of two‐dimensional materials. Quantum confinement (number of layers) and defect engineering enables their properties to be tuned over a broad range, including metal‐to‐semiconductor transitions, magnetic ordering, and topological surface states. They possess various polytypes, often of similar formation energy, which can be accessed by selective synthesis approaches. They excel in mechanical, optical, and chemical sensing applications, and feature long‐term air and moisture stability. In this Minireview, we summarize the recent progress in the field of noble‐metal chalcogenides and phosphochalcogenides and highlight the structural complexity and its impact on applications.

## Graphical abstract

**The noble art**: Noble‐metal dichalcogenides and phosphochalcogenides are a novel class of two‐dimensional materials. This Minireview summarizes the progress in their synthesis, characterization, and application, highlighting their unique structural features and relationships.

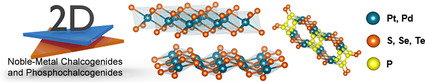

## 1 Introduction

Noble‐metal chalcogenides (NMCs) have been known since the 19th Century.^1^, ^2^ In contrast, their dichalcogenide forms (NMDCs: MX_2_, M=Pd, Pt, X=S, Se, Te) were well‐characterized only in the 20th Century by the groups of Grønvold^3^, ^4^, ^5^, ^6^, ^7^ and Hulliger.^8^, ^9^ Many NMDCs, for example, PtSe_2_ and PdTe_2_,^10^ and also some of the less‐well‐known noble‐metal phosphochalcogenides (NMPCs),^11^, ^12^, ^13^, ^14^ are layered materials. However, interest in these has been limited because of their high cost, and little research has been carried out since the 1960s. This changed, however, with the “rediscovery” of NMDCs in 2014^15^ as two‐dimensional (2D) materials, since only one or few layers are required for application in 2D devices, thus removing the economic bottleneck and boosting research efforts in the field.

NMDCs gained a lot of attention as new members of the 2D family, since they showed a layer‐controllable metal‐to‐semiconductor transition,^16^, ^17^ with few layers and the bulk phase featuring the topological properties of Dirac type‐II semimetals.^18^, ^19^, ^20^, ^21^ The NMDCs also showed an outstanding performance as sensors, for example, for pressure or for specific molecular species.^22^, ^23^, ^24^, ^25^, ^26^ Their chemistry, which includes highly anisotropic structural features,^27^ low‐energy differences between different polymorphs,^28^ controllable phase changes,^29^, ^30^, ^31^, ^32^ and catalytic properties,^17^, ^33^, ^34^, ^35^, ^36^ differs from that of the transition‐metal dichalcogenides (TMDCs), such as MoS_2_ and WSe_2_.^37^ This is caused by electron correlation and relativistic effects, which render their theoretical investigation challenging. To allow for direct comparison, this Minireview contains a recalculation of the structural, vibronic, and electronic features of these materials on the basis of density‐functional theory (DFT; see the Supporting Information for details). This allows a consistent comparison between theory and experiment, in particular for phase stabilities and vibrational spectra. We provide an extensive characterization of NMDCs and NMPCs, highlighting recent developments in the field. A detailed review focusing on the application of NMDCs was recently published by Pi et al.^38^ and an in‐depth analysis of mechanical and photocatalytic properties of NMDC monolayers was reported by Xiong et al.^39^ Here, we put an emphasis on the phase stabilities and transitions of the NM(D/P)Cs, which define if and how single or few layer materials can be obtained in experiments.

## 2 Structures of Layered NM(D/P)Cs

The palladium and platinum dichalcogenides (PdX_2_ and PtX_2_) were first synthesized and characterized in the early 1900s.^2^, ^3^, ^4^, ^5^, ^6^, ^7^, ^40^ They feature rich polymorphism, in accord with a complicated phase diagram.^7^ Before we turn to recent advances in their synthesis as 2D materials, we discuss their structural prototypes, stabilities, and phase relationships.

**Figure 1:**
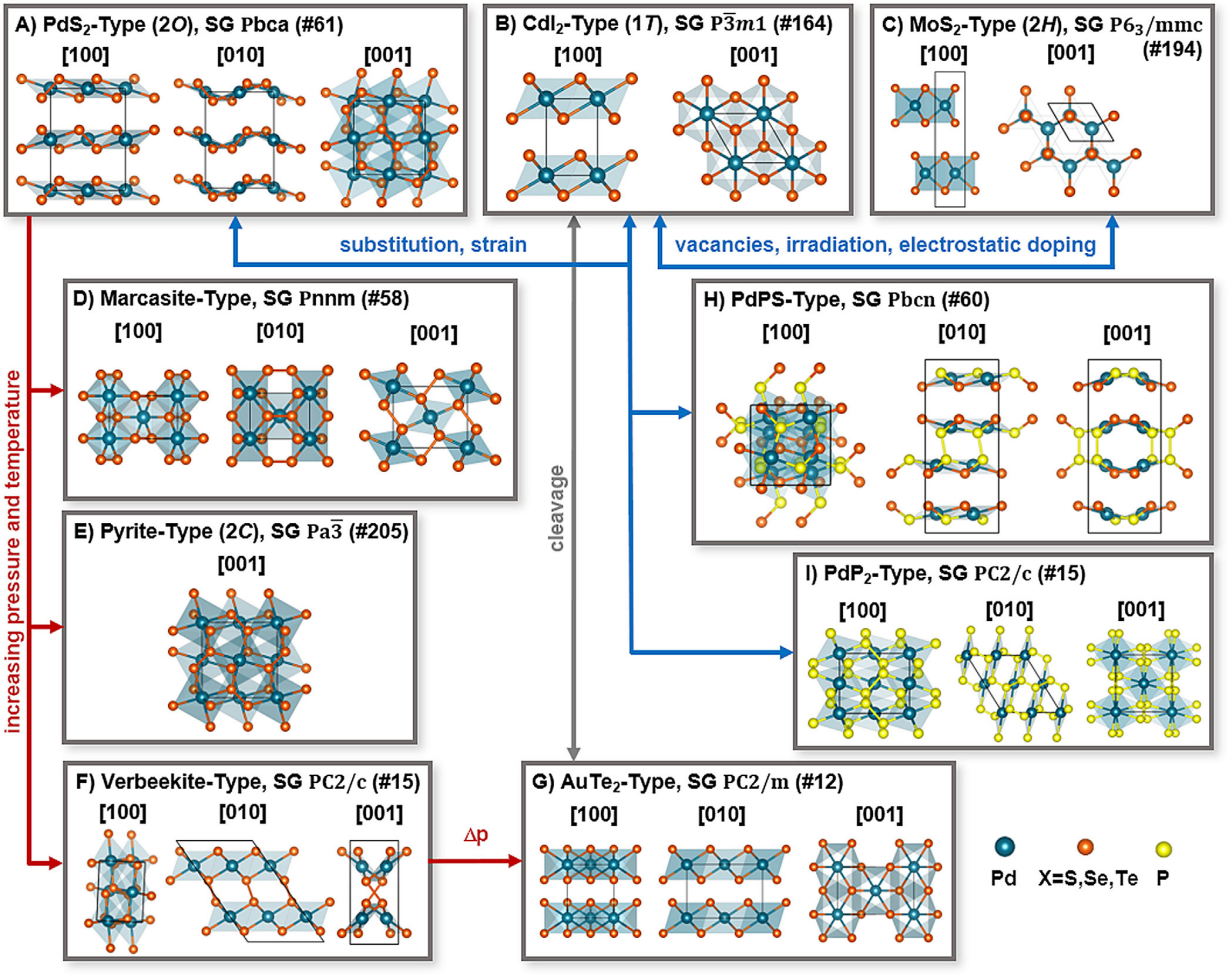
Overview of the main structural prototypes of the NM(D/P)Cs. Commonly used abbreviations, the space group (SG), and the SG number (#) are given in parentheses. Structures are shown along the directions indicated in brackets. Possible means to achieve phase transitions are shown with arrows.

PtX_2_ favors the CdI_2_‐type structure, which is common to Group 4, and is referred to as the 1*T* phase (Figure 1 b).^4^, ^36^, ^41^ For TMDCs of Groups 5 and 6, the stability of the MoS_2_ type (called the 2*H* phase, Figure 1 c) is higher, which can be related to the increasing number of d electrons.^42^, ^43^, ^44^ For TMDCs with larger numbers of d electrons, the 2*H* phase is typically unstable.^43^

For PtS_2_, we determine the 2*H* phase to be unstable, whereas for PtSe_2_, we obtain a large energy difference of 144 kJ mol^−1^ (all energies are given per formula unit) to the 1*T* phase. Nonetheless, 2*H*‐PtSe_2_ has been observed experimentally in few‐layer nanosheets by Wang et al.,^30^ and Lin et al.^29^ have shown a controllable process to obtain mixed 1*T*‐2*H*‐PtSe_2_ structures. These findings illustrate that nanoscale processes can be used to access these high‐energy structures.

In contrast, we predict much smaller energy differences of less than 58 kJ mol^−1^ between the different polymorphs of PdX_2_ (Table 1). In recent publications, these relatively small differences have led to some controversy in the prediction of the most stable phase when there are only a few layers.^15^, ^28^, ^46^, ^63^ Experimentally, PdTe_2_ also prefers the 1*T* phase,^54^, ^64^ whereas both PdS_2_ and PdSe_2_ adopt a completely different layered structure with an orthorhombic unit cell, here called the 2*O* phase (Figure 1 a).^3^, ^5^, ^8^, ^45^, ^49^, ^52^ This structure is quite surprising as a result of its unique pentagonal tiling.^48^ Whereas the 2*H* and 1*T* prototypes, as well as their differently stacked (3*R*) and distorted variants (1*T*′, *T*_d_, AuTe_2_)^31^, ^44^ are similar to each other, the 2*O* structure seems to be special.

**Table 1:** Chemical formula, structure prototype and relative stability, lattice parameters, band gaps, reported synthesis, and selected properties and applications of layered NM(D/P)Cs. Entries marked with an asterisk (*) refer to theoretical predictions from this work or from the given citation.

Formula	Structure and rel. stability	Lattice constants [Å]	Band gap [eV]	*E*_exfl._ [J m^−2^]	Synthesis	Properties and applications
PdS_2_	PdS_2_ 3	*a*=5.460 *b*=5.416 *c*=7.531	0.7^8^ (indirect)	0.31*	chemical vapor transport^45^	superconductivity at ca. 16 GPa^45^
	CdI_2_* (+44.4 kJ mol^−1^)	*a*=*b*=3.483* *c*=5.226*	metallic*)	0.32*	–	single‐material logic junctions^46^
						
PdSe_2_	PdS_2_ 3	*a*=5.741 *b*=5.866 *c*=7.691	0.5^47^ (indirect)	0.33*	self‐flux method^48^ chemical vapor deposition^27^	high mobility FETs^49^ polarization‐sensitive photodetectors^26^
	marcasite^50^ (+44.8 kJ mol^−1^)	*a*=4.873 *b*=6.013 *c*=3.930	metallic*		high pressure/high temperature, e.g. in diamond anvil cells	–
	pyrite^50^, ^51^ (+38.6 kJ mol^−1^)	*a*=*b*=*c*=6.100	metallic^52^			superconductivity at 23 GPa^52^
	verbeekite^53^ (+1.4 kJ mol^−1^)	*a*=10.928 *b*=4.154 *c*=6.710 *β*=125°	0.85* (direct)*			–
	CdI_2_* (+31.7 kJ mol^−1^)	a=*b*=3.734* *c*=4.889*	metallic*	0.40*	–	single‐material logic junctions^46^
						
PdTe_2_	CdI_2_ 54	*a*=*b*=4.037 *c*=5.126	metallic^8^	0.85*	molecular beam epitaxy^55^, ^56^	Dirac type‐II fermions and superconductivity^18^, ^19^, ^21^, ^56^
	pyrite* (+13.6 kJ mol^−1^)	*a*=*b*=*c*=6.561	metallic*		–	–
						
PtS_2_	CdI_2_ 4, 8	*a*=*b*=3.543 *c*=5.039	0.7^8^ (indirect)	0.20*	vapor assisted conversion^57^	high‐gain phototransistors^58^ chemical sensors^57^
						
PtSe_2_	CdI_2_ 4	*a*=*b*=3.728 *c*=5.081	metallic	0.51*	epitaxial growth^17^ thermally assisted conversion^24^	chemical sensors^22^, ^24^ piezoresistive sensors^23^
	MoS_2_ (+162 kJ mol^−1^)	*a*=*b*=3.507* *c*=11.334*	metallic*	1.35*	chemical vapor deposition^30^	–
						
PtTe_2_	CdI_2_ 4	*a*=*b*=4.026 *c*=5.221	metallic^8^	0.28*	self‐flux method^59^	Dirac type‐II fermions^19^, ^59^
	MoS_2_ (+100 kJ mol^−1^)	*a*=*b*=3.879 *c*=11.957	metallic*	0.76*	–	–
						
PdPS	PdPS^13^	*a*=13.304 *b*=5.678 *c*=5.693	0.65^10^, ^14^ (indirect*)	0.31*	elementary reaction^14^	photocatalysis^60^
						
PdPSe	PdPS^10^, ^14^	*a*=13.569 *b*=5.824 *c*=5.856	0.15^10^, ^14^ (direct*)	0.33*	elementary reaction^14^	photocatalysis^60^
						
PtTe	PtTe	*a*=6.860 *b*=3.960 *c*=7.044 *β*=109°	metallic*^61^	0.95*^61^	elementary reaction^62^	electrocatalysis^61^

To provide insight into the stability and properties of these phases, we show that the structure of PdS_2_ and PdSe_2_ (Figure 1 a) is related to the pyrite‐type structure (Figure 1 e).^8^ We then connect the pyrite structure to its high‐pressure phases (marcasite^50^ and verbeekite^53^ in Figure 1 d,f) and show its transformation back to the 1*T* phase (Figure 1 b).

In the next step, we turn to even more chemically complex systems, namely those containing substitutions and defects. We show similarities between the 2*O* phase and the structure of PdPS (Figure 1 h), which occurs as an intermediate to the PdP_2_ structure (Figures 1 i).

### 2.1 Relationship of the PdS_2_‐Type and CdI_2_‐Type

**Figure 2:**
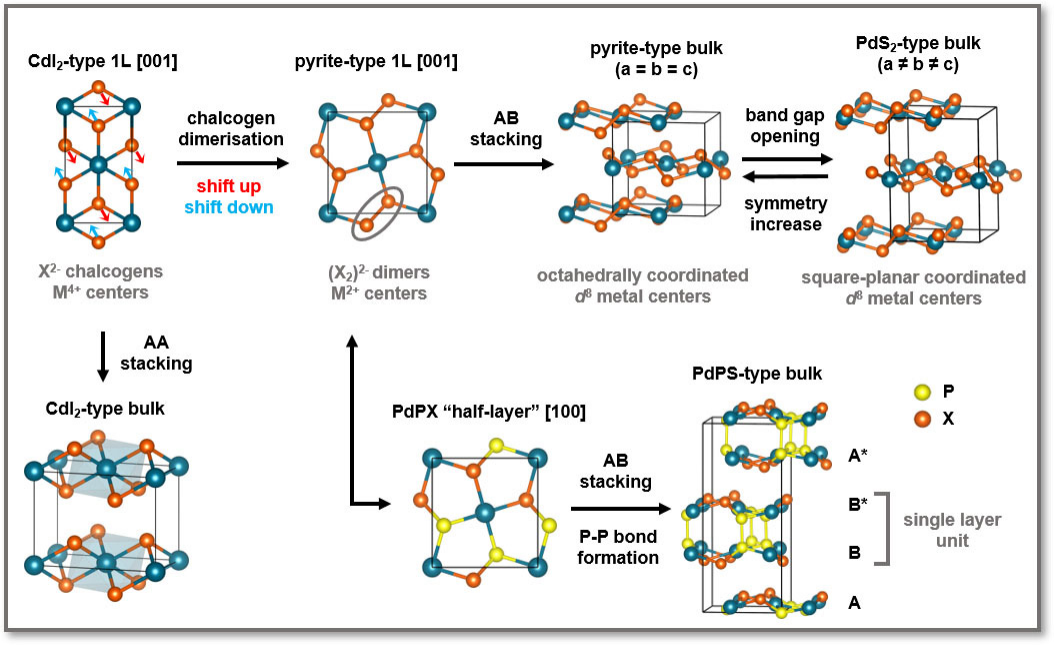
Structural relationships between the PdS_2_ and PdPS structures. The CdI_2_ prototype can be transformed via the pyrite‐type to the PdS_2_‐type structure, and the pyrite structure is the parent for the PdPS structure. Note that the PdPS structure consists of six atomic layers, two of them are connected by phosphorus bonds. Layers that are antisymmetric with respect to each other are indicated with an asterisk (*).

**Figure 3:**
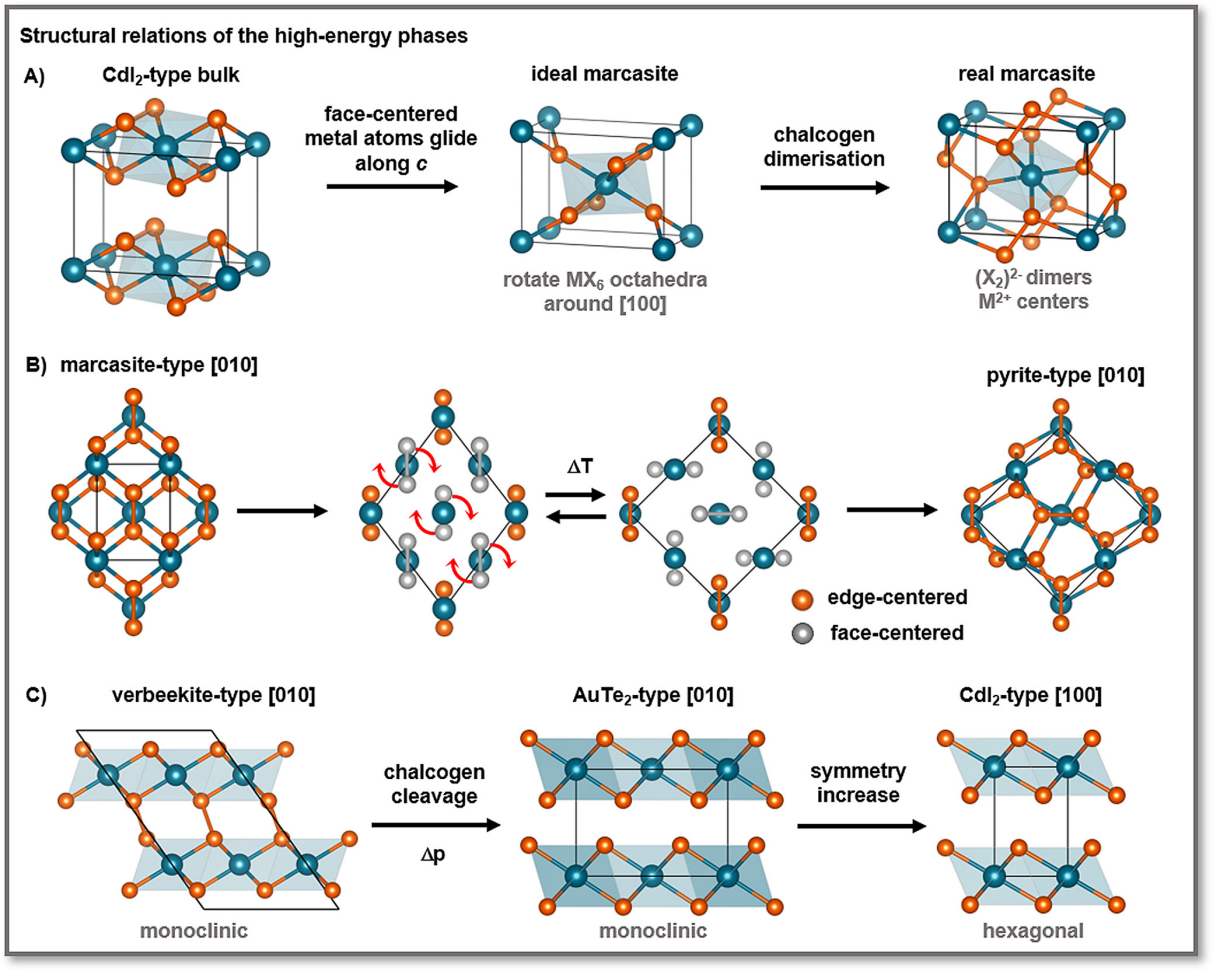
A) Relationship of the 1*t* prototype to the marcasite prototype, B) relationship of the marcasite prototype to the pyrite structure, and C) relationship of the verbeekite and 1*T* structures.

Simple concepts, such as oxidation state and charge distribution, help to identify the key factors for the structural stability of these polymorphs.^42^ According to the ionic counting scheme, the metal centers in the octahedrally coordinated 1*T* phase formally have an oxidation state of +IV.^64^ PtX_2_ with this oxidation state is stable due to the involvement of f orbitals, whereas PdX_2_ prefers to lower the oxidation state to +II. This can be achieved by pairing the chalcogen atoms to form (X_2_)^2−^ dimers, thereby leading to the pyrite‐ and marcasite‐type structures, both with octahedral coordination of the metal centers (Figures 2 and 3 a). Phase transitions between the 1*T*, marcasite, and pyrite structures have been observed in IrTe_2_ 80 and CoTe_2._ 65

We support the validity of these simple chemical descriptors with the Hirshfeld charge analysis (see Figure S1 in the Supporting Information). There, we show a large charge transfer (corresponding to a higher oxidation state) in the 1*T* structures of PdS_2_ and PdSe_2_ compared with a smaller charge transfer (corresponding to a lowered oxidation state) in the pyrite‐like phases. For PdTe_2_, the charge transfer between the 1*T* and pyrite‐phases has a similar magnitude, thus indicating no change in the oxidation state. Since formation of the Te‐Te dimers is less likely, PdTe_2_ prefers the 1*T* phase.

The ground‐state structure of PdS_2_ and PdSe_2_ has a lower symmetry than the pyrite prototype to avoid the d^8^ electron configuration in the octahedral crystal field. Therefore, one axis is elongated, imposing a change to a square‐planar coordination. This leads to a novel structure with AB‐stacked, buckled layers, which can be expressed by the sum formula Pd^2+^(X_2_)^2−^. Furthermore, one lattice vector (either *a* or *b*) is slightly elongated to avoid degenerate d states on the metal centers. Consequently, the PdS_2_‐type structures are generally semiconductors,^8^ but close their band gaps under pressure.^45^, ^51^, ^52^

### 2.2 High‐Energy Structures

Under pressure, the PdS_2_‐type structure reversibly transforms to the pyrite‐type structure.^45^, ^50^, ^51^, ^52^, ^53^, ^66^ The pyrite‐phase competes with the marcasite‐type structure, as it is close in energy (Δ*E*=6.2 kJ mol^−1^ for PdSe_2_). This is well‐known for these two phases, since they differ mainly in the orientation of the chalcogen dimers (Figure 3 b).^65^ Larchev and Popova^50^ observed the marcasite phase of PdSe_2_ at 7.5 GPa and below 900 °C, whereas the pyrite‐phase was preferred above this temperature.

Other phases with reduced interlayer distances can occur between these phases.^66^ In the case of PdS_2_, much higher pressures (>16 GPa) are needed to obtain the pyrite phase.^45^ Pyrite‐PdS_2_ becomes superconducting at a critical temperature of 8 K at 37.4 GPa,^45^ while pyrite‐PdSe_2_ requires 13.1 K and 23 GPa.^52^

So far, the synthesis of PdTe_2_ in the pyrite phase has not been reported. Under a pressure of 5 GPa at 300 °C, Soulard et al.^64^ observed a continuous phase transition, where the interlayer Te−Te bond distances become shorter than the intralayer Pd−Te bond distances. This indicates that Te‐Te dimers could be formed at elevated temperatures and pressures and may lead to pyrite‐PdTe_2_.

To date, there are also no reports on the synthesis of PdS_2_ or PdSe_2_ in the 1*t* phase. The structural element that decides whether the 1*T* phase is more stable than the 2*o* phase is the chalcogen dimers. The chalcogen states are also the dominating states just below the Fermi level, as confirmed by the atom‐projected density of states (Figures S2–S4). Hence, we expect that laser irradiation or electrostatic doping, as shown for few‐layer MoTe_2_,^67^ would be promising approaches to induce a phase transformation. From their structural prototypes, we conclude that uniform pressure is unlikely to transform 2*O*‐PdS_2_/Se_2_ into 1*T*‐PdS_2_/Se_2_, because the latter have larger cell volumes.

An indirect approach to obtain 1*t*‐PdSe_2_ via a high‐energy phase has been suggested by Lei et al.:^31^ At a temperature of 1600 K and a pressure of 11.5 GPa, 2*o*‐PdSe_2_ undergoes a phase transition to the verbeekite structure (Figures 1 f and 3 c).^53^ Verbeekite‐PdSe_2_ was first found as a mineral^68^ and appears to be a stable polymorph of PdSe_2_ (Δ*E*≈1.4 kJ mol^−1^), which is closer to the structure of PdP_2_ (Figure 1 i)_._ 12, 53 Lei et al.^31^ predict that verbeekite‐PdSe_2_ may transform under pressure to the slightly distorted 1*t* structure known as AuTe_2_‐type (Figure 3 c) and few‐layer 1*T*‐PdSe_2_ may then be cleaved from this phase.

### 2.3 Ternary Metal Chalcogenides, Phosphides, and Beyond

In addition to pressure and temperature, substitution and defects also lead to unique structural motifs of the palladium chalcogenides. Hulliger^8^, ^10^ showed that the ternary mixture PdSSe remains in the PdS_2_‐type, whereas PdSeTe already occurs in the CdI_2_‐type. This indicates that a mixture of PdSe_1+*x*_Te_1−*x*_ for 0<*x*<1 exists, where the 1*T* phase becomes more stable because the formation of chalcogen–chalcogen dimers is no longer favorable.

On the other hand, the ternary mixtures PdPS and PdPSe adopt a novel, layered structure, which is an intermediate between the PdP_2_ and PdS_2_ structures.^10^, ^13^ These mixtures feature the PdS_2_‐type stacking, but have additional covalent interlayer P−P bonds (Figure 2).

Lastly, introducing chalcogen vacancies in PdSe_2_ is interesting for phase transformations and resistive‐switching memory devices, because such defects have low diffusion barriers.^69^ Many selenium vacancies can lead to an “interlayer fusion”, as shown by Lin et al.^70^ The resulting material has a new layered crystal structure, with the sum formula Pd_2_Se_3_, which could be an interesting component in heterojunctions.^71^ In PtTe_2_, removing half of the chalcogen atoms to obtain PtTe yields a different layered structure with metallic monolayers.^61^

## 3 Synthesis

The first synthetic approaches to obtain bulk NM(D/P)Cs required reactions at high temperatures over the course of several weeks.^3^, ^4^, ^5^, ^51^ The synthesis methods for NM(D/P)C materials have been strongly improved in recent years, when these materials regained scientific interest as a consequence of their layered bulk forms. Thin layers of these materials can be accessed by alternative synthesis approaches, such as chemical vapor deposition (CVD) or molecular beam epitaxy (MBE).

In 2015, PtSe_2_ monolayers were successfully grown as high‐quality, single‐crystalline films by Wang et al.,^17^ who showed that a low growth temperature of 270 °C was needed to directly selenize a Pt(111) surface. As a result of the necessary ultrahigh vacuum and a difficult transferring process, Dong and co‐workers^30^ proposed an alternative method: CVD based on H_2_PtCl_6_ and selenium precursors in the temperature range 300–900 °C on a sapphire substrate. This approach results in PtSe_2_ nanosheets, which are easier to transfer to a poly(methyl methacrylate) substrate. Both approaches can yield the metastable 2*H*‐PtSe_2_ form.^29^

A more scalable approach was reported by Yim et al.,^24^ who employed thermally assisted conversion on a Si/SiO_2_ substrate. This method is remarkable due to the low growth temperature of 400 °C, which allows polycrystalline PtSe_2_ films with nanometer‐sized grains to be obtained^23^ in a fashion that is directly compatible with back‐end‐of‐line semiconductor processing.

These approaches are, however, more difficult to apply to PtS_2_ due to the competing nonlayered PtS phase.^57^, ^72^ Monolayer PtS_2_ has been obtained by mechanical exfoliation,^73^ which limits the flake size to small lateral dimensions. Xu et al.^57^ reported the growth of wafer‐scale PtS_2_ films, but controlling the surface morphology and thickness is more challenging because of the need to convert the PtS phase into PtS_2_ by varying the sulfur vapor pressure during annealing.^72^ The same challenge occurs with PdS_2_,^5^ which has a different structure and has not yet been exfoliated. Single‐crystalline bulk PdS_2_ can be obtained by chemical vapor transport.^45^

In 2017, 9 nm thick films of 2*O* PdSe_2_ were successfully obtained by Chow et al.^49^ by a self‐flux method at 850 °C over the course of 50 h. Oyedele et al.^48^ used the same synthesis approach and managed to mechanically cleave monolayers from the as‐grown material. In both cases, the unique pentagonal Cairo‐tiling of this structure was maintained.^48^ In 2019, the controllable CVD growth of 2*O*‐PdSe_2_, ranging from 2 to 20 layers on gold foil, was reported by Jiang et al.^27^ Highly anisotropic ribbons were formed, as a result of the orthorhombic crystal structure.

Bulk materials of the tellurides have been synthesized,^4^, ^54^, ^74^ but few‐layer PtTe_2_ and PdTe_2_ are difficult to obtain. Li et al.^55^ used MBE to grow PdTe_2_ with various thicknesses down to four layers on bilayer graphene. Liu et al.^56^ employed MBE on a SrTiO_3_(001) surface to obtain PdTe_2_ between 1 to 20 layers, but more scalable approaches still need to be developed.

In general, all of the layered NMDCs have exfoliation energies that allow for mechanical exfoliation (*E*_exfl._<1 J m^−2^, Table 1). Some of them, for example, PdTe_2_, are clearly much harder to exfoliate than, for example, graphene. We want to highlight the possibility of exfoliating the NMPCs (Table 1), which have not been exfoliated to date.

## 4 Properties and Applications

### 4.1 Platinum Dichalcogenides

#### 4.1.1 Electronic Properties

The PtX_2_ materials excel in optoelectronic applications and feature strongly layer‐dependent properties. The bulk materials range from semiconducting for PtS_2_ (band gap of 0.7 eV)^8^ to metallic for PtTe_2_.^8^ Hulliger measured a band gap of PtSe_2_ of 0.1 eV from its resistivity, while theoretical investigations predicted semimetallic behavior.^15^, ^75^ In 2015, Wang et al.^17^ confirmed that the bulk material is semimetallic by angle‐resolved photoemission spectroscopy (ARPES) measurements. The authors confirmed the predictions^15^ of a semimetal‐to‐semiconductor transition at the monolayer limit, with a band gap of about 1.2 eV. This fascinating effect was supported by Ciarrochi et al.,^16^ and later‐on Shi et al.^34^ determined this transition to already happen at three layers. These observations agree with our calculations of the band structure (Figures S5–S9). Additionally, our calculations show a semimetal‐to‐semiconductor transition from bilayer to monolayer PtTe_2_, with a significant influence of spin–orbit coupling (SOC) on the band structure.

Besides through the number of layers, the electronic properties of PtSe_2_ can be tuned through strain and defect engineering. Whereas defect‐free PtSe_2_ is diamagnetic, a transition to a half‐metallic ferromagnetic state for monolayer PtSe_2_ at a critical strain of 5 % was predicted,^76^ which favors the formation of single Pt vacancies and may cause the p‐type conductance in PtSe_2._ These point defects occur intrinsically in ultrathin 1*T*‐PtSe_2_ layers,^77^ and Avsar et al.^78^ verified the emergence of magnetism as a result of vacancies in ultrathin metallic PtSe_2_ both experimentally and theoretically. In addition, the authors showed that the layer number determines whether the ground‐state ordering is ferromagnetic or antiferromagnetic. The possibility to engineer the magnetic properties of PtSe_2_ through defects renders it very interesting for spintronics.

Lastly, exotic new electronic states can be realized in this family of materials by making use of SOC. Huang et al.^19^ predicted that the P$\bar{3}$ *m*1 symmetry enables the existence of stable, strongly tilted type‐II Dirac fermions in 1*T*‐PtSe_2_, which have been experimentally verified in 1*T*‐PtTe_2_.^59^ These features might be the cause of the anomalous magnetotransport in PtSe_2_ microflakes,^79^ but require further investigation in the context of defect‐ and strain‐induced magnetism.

#### 4.1.2 Applications

The layer‐sensitivity of the electronic properties is intriguing for nanoelectronic and sensing applications. Bulk PtS_2_ and PtSe_2_ feature p‐type transport with Seebeck coefficients of 500 μV^−1^ K^−1^ and 40 μV^−1^ K^−1^, respectively.^8^ A few‐layer PtS_2_ field‐effect transistor (FET) shows mobilities exceeding 62.5 cm^2^ V^−1^ s^−1^ 80 and a few‐layer PtSe_2_‐FET featured even higher room‐temperature mobilities of 210 cm^2^ V^−1^ s^−1^.^81^

Yim et al.^24^ built a chemical gas sensor for NO_2_ adsorption with ultrafast response times and high sensitivity: After selenizing 0.5 nm Pt by thermally assisted conversion, which led to thicknesses between 3 and 5 layers,^82^ the sensor responded to a 100 sccm flow of a NO_2_/N_2_ gas mixture for 10 s, with the original resistance being fully recovered in a pure N_2_ flow at room temperature. The authors determined response and recovery times of 2.0 to 53.7 s and 7.4 to 38.7 s, respectively, at 0.1–1.0 ppm NO_2_, with a limit of detection below 100 ppb. The superior gas‐sensing properties of monolayer PtSe_2_ have been theoretically verified by Sajjad et al.,^22^ hence, controlling the thickness of the PtSe_2_ films down to the monolayer limit may improve the performance of the sensor. PtS_2_ features even lower detection limits of 0.4 ppb for NO_2_, but with lower sensitivity and higher response/recovery times.^57^

Based on 4 nm thick PtSe_2_ films, Yim et al.^24^ built PtSe_2_/n‐Si Schottky diodes with a maximum responsivity of 490 mA W^−1^ at a wavelength of 920 nm. The photoresponsivity of PtSe_2_ in the infrared region allows its usage as an absorber with tunable sensitivity.^83^ The mid‐infrared sensitivity is very promising for optoelectronics and could be enhanced by defect‐engineering,^84^ and the small band gap of 0.21 eV in a bilayer PtSe_2_ combined with fast carrier dynamics could be exploited for saturable absorbers to generate ultrafast laser pulses.^85^ Zeng et al.^86^ reported PtSe_2_/GaAs heterojunction photodetectors with peak sensitivity between 650 and 810 nm, where the on/off response speed of 5.5/6.5 μs is the highest among other Group 10 TMDCs. The performance of PtS_2_ on Al_2_O_3_/Si substrates as a photodetector is lower (300 A W^−1^ at 830 nm),^80^ but PtS_2_ on h‐BN reaches 1560 A W^−1^ at 500 nm.^58^ The striking advantage of PtS_2_‐ and PtSe_2_‐based photodetectors is their much lower response/recovery times compared, for example, to MoS_2_ (4 s/9 s).^87^

The large response of the electronic properties of PtSe_2_ to strain can be exploited for optical and mechanical sensors.^88^ Du et al.^89^ showed that a compressive strain of 3 % induces a semiconductor‐to‐semimetal transition in a monolayer PtSe_2_, which is easier to achieve than in MoS_2_ because of the low in‐plane stiffness of 64 N m^−1^.

Few‐layer PtSe_2_ is semimetallic and the density of states at the Fermi level increases under strain, thereby giving rise to a large piezoresistive effect. This allowed Wagner et al.^23^ to construct highly sensitive pressure sensors based on 4.5 nm thick PtSe_2_ membranes. The sensitivity of these devices was an order of magnitude higher (5.51×10^−4^ mbar^−1^) than those of other nanomaterial‐based devices, including graphene, with a negative gauge factor up to −84.8. Boland et al.^90^ advanced that approach by growing the PtSe_2_ layers directly on top of flexible polyimide foils suitable for high‐frequency applications. By patterning the PtSe_2_/polyimide membranes in a three‐dimensional kirigami fashion, Okogbue et al.^91^ built FETs with device stretchability above 2000 % as well as tunable electrical conductance and photoresponsivity.

In terms of catalysis, Chia et al.^36^ determined that the catalytic activity of platinum dichalcogenides for the hydrogen evolution reaction (HER) increases with the size of the chalcogen atoms. PtTe_2_ outperforms PdTe_2_ as a result of the lower overpotential barrier of 0.54 eV versus the reversible hydrogen electrode (RHE) and also because it is less prone to tellurium stripping.^35^ The performance of the bulk material towards the HER is reasonable, but can be enhanced by increasing the amount of edge sites^92^ or by reducing the bulk thickness. Shi et al.^34^ indicated atomically thin 1*T*‐PtSe_2_ to be a perfect electrocatalyst, with a record high HER efficiency comparable to the traditional Pt catalysts.

### 4.2 Palladium Ditelluride

#### 4.2.1 Characterization

For PtX_2_, Raman spectroscopy has been shown to be a powerful method to monitor the layer thickness.^41^ For PdTe_2_, we predict a sizeable shift of about 50 cm^−1^ of the E_g_ mode in going from the bulk phase to a monolayer (Figure S10). Li et al.^55^ reported the growth of four‐layer PdTe_2_ by molecular beam epitaxy, and measured the Raman spectrum for six layers, which showed a shift in the E_g_ mode by about 7 cm^−1^ to higher wavenumbers, whereas the A_1g_ mode remained unchanged.

As a consequence of the in‐plane conductivity of PdTe_2_, we largely overestimate the intensity of the in‐plane E_g_ mode relative to the out‐of‐plane A_1g_ mode. Prediction of the Raman spectra of metallic systems is challenging and will require further investigations. For comparison with experiments, we indicate the position of the out‐of‐plane A_1g_ mode in gray (Figure S10). Our calculated bulk frequencies are in excellent agreement with the experiments of Fei et al.,^18^ hence, we expect valid predictions for fewer layers. In this case, the influence of layer–layer coupling may lead to the appearance of new modes which are lower in symmetry than the E_g_ and A_1g_ bulk modes. These modes might be used to precisely identify the layer number, such as the bilayer mode at $\tilde{\nu }$ =250 cm^−1^ for 1*t*‐PdTe_2_.

#### 4.2.2 Electronic Properties

PdTe_2_ is more similar to PtX_2_ than to the other palladium dichalcogenides. Bulk 1*T*‐PdTe_2_ is metallic and diamagnetic^8^ and was predicted to undergo a transition to a narrow‐gap semiconductor (about 0.14 eV) as a monolayer,^56^ whereas our calculations suggest a semimetallic state when SOC is included (Figures S11 and S12). Weak, temperature‐independent paramagnetism (μ≪μ_B_) is usually observed,^93^ which might be caused by defects.^78^ PdTe_2_ features rather strong interlayer interactions, but with a cleavage energy of 0.85 J m^−2^, it should still be exfoliable (Table 1). These interlayer interactions mean it can be considered an intermediate between 2D and 3D materials.^94^ This intermediate state can lead to exotic electronic features, such as spin‐polarized topological surface states, as a result of band inversion and complex spin textures.^95^

The physics resulting from the intriguing electronic and phononic structure of PdTe_2_ are still under debate. PdTe_2_ gained a lot of attention as it was the first material featuring both Dirac fermions and superconductivity,^21^, ^96^, ^97^ which is the precondition for the emergence of Majorana fermions.^98^ A Majorana is an exotic particle, being defined as a fermion that is its own antiparticle. Majoranas can be realized as quasiparticles in condensed matter physics,^99^ and are an important component for the realization of quantum information technology. Experimental realization of Majoranas has been reported only recently.^100^

PdTe_2_ becomes superconducting at temperatures below 2 K.^8^, ^96^, ^101^, ^102^ The symmetry of the states causing the superconductivity is still under debate:^103^ although there is agreement that it is conventional superconductivity,^20^ it remains unclear whether it is of type‐I^102^ or type‐II.^95^ Even though the electronic structure of PdTe_2_ changes greatly with the number of layers, the superconductive state remains robust even down to two layers^56^ and the superconducting surface states are robust under pressures up to 2.5 GPa.^101^

Evidence of topological surface states in PdTe_2_ was found by Liu et al.^96^ through ARPES measurements, but the Dirac point was found at the Γ‐point deep below the Fermi level (−1.75 eV). Noh et al.^21^ found another Dirac cone much closer to the Fermi level (−0.5 eV) at *k*=(0, 0, ±0.4) which features strong tilting in the *k*_z_ direction.^18^, ^102^ Thus, these are type‐II Dirac semimetals, where the momentum‐dependent tilting breaks Lorentz invariance.^59^, ^102^, ^104^ Only type‐II Dirac semimetals allow for Majorana modes, type‐I Dirac semimetals do not.^105^ Under pressures of 4.7–6.1 GPa, the Dirac cone can be tuned from type‐II to type‐I.^106^

Another property of Dirac semimetals is the possibility to break either time reversal symmetry or inversion symmetry, and thus split the doubly degenerate Dirac cone into two separate Weyl nodes.^104^, ^107^ These feature spin‐polarized Fermi arcs as topological surface states, as well as an anisotropic negative magnetoresistivity.^94^, ^108^ Since this intrinsic magnetism could be introduced by defects, PdTe_2_ is an excellent candidate to study Weyl metals and superconductivity at the same time.

### 4.3 Palladium Disulfide and Diselenide

#### 4.3.1 Characterization

The unique structural motif of crystalline 2*O*‐PdS_2_ and 2*O*‐PdSe_2_ allows the number of layers to be precisely determined by Raman spectroscopy, since stacks with an odd number of layers have *P*2_1_/*c* symmetry (with inversion center), whereas stacks with an even number of layers have *Pca*2_1_ symmetry (no inversion center).^109^ Hence, the second harmonic generation occurs only in stacks with an even layer number.^109^ Furthermore, the Raman intensities are highly polarization‐sensitive due to the anisotropic crystal structure.^26^, ^27^, ^109^

**Figure 4:**
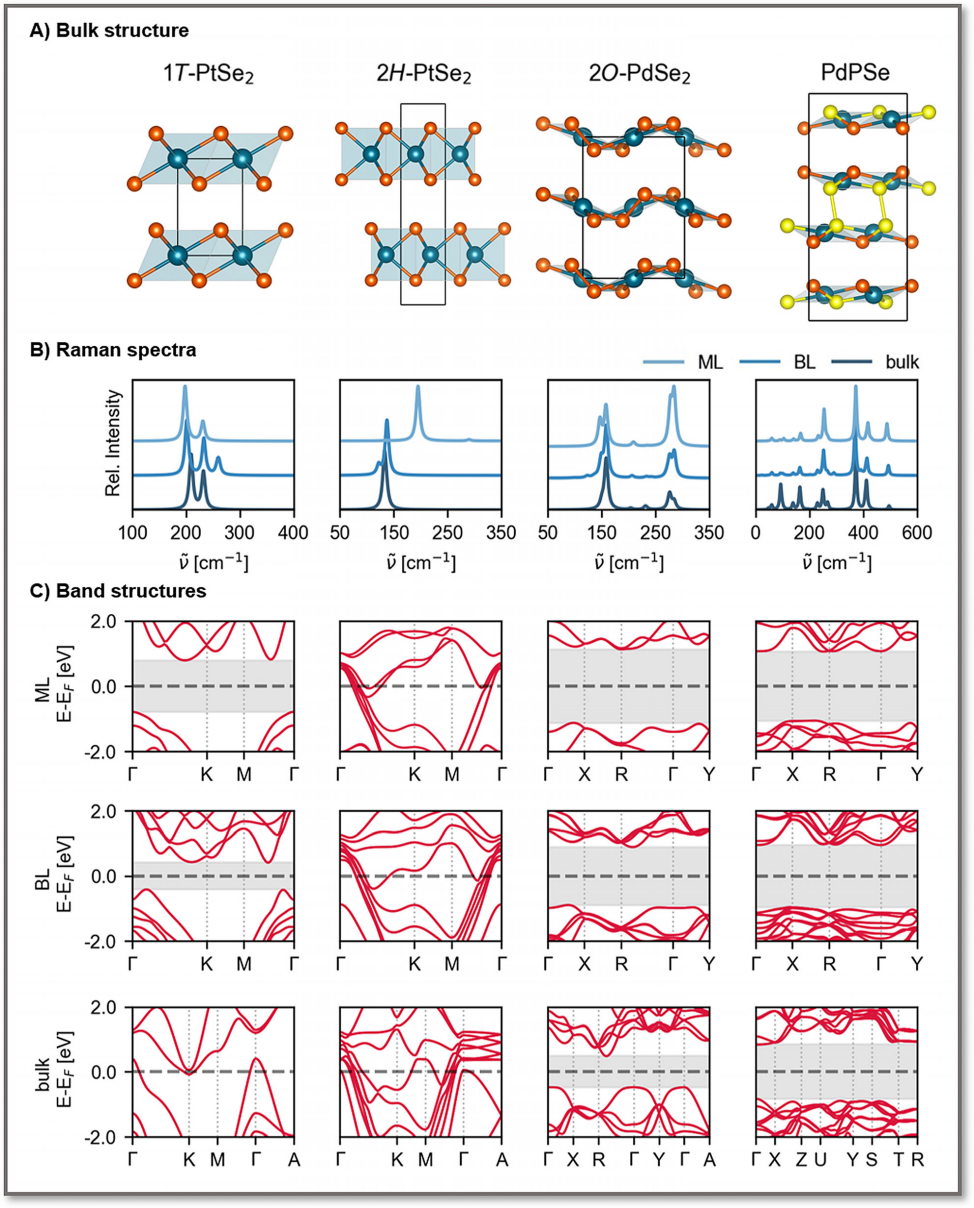
Calculated Raman spectra and band structures for monolayer (ML), bilayer (BL), and bulk polymorphs of some exemplary layered NM(D/P)Cs.

For polycrystalline samples, extracting the number of layers from the Raman spectrum is more difficult because the layer‐dependent wavenumber shifts are small.^48^ We predicted the spectra of monolayer, bilayer, and bulk 2*O*‐PdX_2_ (Figures 4 b as well as Figures S13–S16), where our calculated frequencies are in good agreement with the experimentally observed ones.^48^, ^49^

In the case of 2*O*‐PdSe_2_, the B11g mode increases its splitting to the A1g mode for fewer layers, which can be employed for monitoring the exfoliation progress. We label the modes according to Oyedele et al.^48^ to facilitate comparison to experiments, where the label does not necessarily reflect the symmetry in the case of fewer layers. In the case of 2*O*‐PdSe_2_, our calculations show an increase in the intensity of the A3g /B31g signals relative to the B11g /A1g signals, in excellent agreement with experiment.^48^, ^49^ On the other hand, we generally seem to underestimate the intensity of the bulk A3g /B31g signals, as well as the intermediate A2g and B21g signals.

We complement this section with the Raman and IR spectra of the potential 1*T*‐PdS_2_ and 1*T*‐PdSe_2_ (Figures S15 and S16). However, differentiating them from the 2*O* phase in experiment could be challenging: We predict the bulk 1*T*‐E_g_ mode to appear close to the bulk 2*O*‐B11g /A1g signals and the bulk 1*T*‐A_1g_ signal to be close to the bulk 2*O*‐A2g signal. Hence, if the 1*T* phase occurs only as a minor side phase, it is likely to be overlooked. An indication of the presence of the 1*T* phase could be the large intensity of the 1*T* bulk A_1g_ signal, especially for the semiconducting 1*T*‐PdS_2_. Whereas the A2g and B21g signals of the 2*o* bulk usually have small intensities, a strong signal close to the A2g mode could indicate the 1*T* phase. Furthermore, for fewer layers, the 1*T*‐E_g_ mode shifts significantly, whereas the 2*O* modes do not.

#### 4.3.2 Electronic Properties

Bulk 2*o*‐PdS_2_ is a semiconductor with an indirect band gap of about 0.7 eV.^8^ We predict an increase of about 1 eV when going from the bulk phase to a monolayer. Wang et al.^28^ calculated a band gap of 1.6 eV for the monolayer, as well as an anisotropic in‐plane stiffness of 58 N m^−1^ in the *x* direction and 82 N m^−1^ in the *y* direction, and large hole mobilities up to 339 cm^2^ V^−1^ s^−1^.

In the case of PdSe_2_ in the 2*O* phase, there has been some recent controversy regarding the band gap of the bulk material.^48^, ^110^, ^111^ Hulliger^8^ determined a small band gap of 0.4 eV through resistivity measurements. Several theoretical studies predicted a (semi)metal‐to‐semiconductor transition for bulk 2*O*‐PdSe_2_ as a result of the underestimation of the band gap with the PBE functional.^48^, ^110^, ^112^ This led to misinterpretations of optical absorbance spectra yielding a zero band gap for bulk PdSe_2_.^48^, ^86^ However, Chow et al.^49^ clearly proved 9 nm thick 2*O*‐PdSe_2_ to be a semiconductor by integrating it into a FET and showing that the band gap could be closed by an external electric field. Zhang et al.^47^ measured a band gap of 0.5 eV for bulk PdSe_2_. Li et al.^113^ measured a bilayer band gap in the range of 1.15–1.35 eV, with the gap depending on the growth substrate as a result of the proximity screening effect.^114^ In monolayers, a band gap of 1.37 eV was measured.^47^ This is in line with our hybrid functional calculations, where we predict an increase in the band gap from 0.96 eV for the bulk phase to 1.8 eV for the bilayer and to 2.3 eV for the monolayer (Figure 4 c and Figures S17 and S18).

Although neither 2*O*‐PdS_2_ nor 2*O*‐PdSe_2_ undergo metal‐to‐semiconductor transition, this has been predicted for the corresponding 1*T* phase.^15^, ^46^ We calculate a band gap of 0.54 eV and 1.7 eV for bulk phase and monolayer 1*T*‐PdS_2_, respectively, and a semimetal‐to‐semiconductor transition in 1*T*‐PdSe_2_ with a monolayer band gap of 0.89 eV (Figures S17 and S18). These band gaps are about 0.5 eV larger than the ones calculated with PBE.^15^ Such a metal‐to‐semiconductor transition could be employed in single‐material logical junctions with strongly suppressed Schottky barriers.^46^

#### 4.3.3 Applications

Both 2*O*‐PdS_2_ and 2*O*‐PdSe_2_ feature desirable properties for thermoelectric applications and transistors: Hulliger^8^ measured Seebeck coefficients of 240 and 500 μV^−1^ K^−1^ for bulk 2*O*‐PdS_2_ and 2*O*‐PdSe_2_, respectively. Chow et al.^49^ obtained a large total mobility of about 216 cm^2^ V^−1^ s^−1^ in 2*O*‐PdSe_2_‐based FETs after annealing, with an on/off ratio of 10^3^. A key advantage of these materials is their air and moisture stability, even as monolayers.^48^ Very recently, bilayer PdSe_2_ has also been grown by Jiang et al. through CVD.^27^

The optical properties of PdSe_2_ render it highly advantageous for sensing applications and photocatalysts.^39^ Long et al.^25^ reported a highly sensitive, air‐stable, long‐wavelength infrared photodetector based on a MoS_2_/PdSe_2_ heterojunction, which has a high photoresponsivity of 42.1 A W^−1^ at 10.6 μm. The specific detectivity (D*) of this device is as high as 8.21×10^9^ Jones, which is an order of magnitude higher than platinum diselenide (ca. 7×10^8^ Jones)^84^ and graphene (ca. 8×10^8^ Jones).^115^

At shorter wavelengths of 780 nm, Zeng et al.^111^ obtained a photoresponsivity of 300.2 mA W^−1^ with a PdSe_2_‐based photodetector decorated with black phosphorus quantum dots, with low rise and fall times of 38 and 44 μs, respectively. Wu et al.^26^ built a graphene/PdSe_2_/germanium heterojunction with a high polarization sensitivity for near‐infrared imaging (0.2 to 3.04 μm) with a responsivity of 691 mA W^−1^ and rise/fall times of 6.4/92.5 μs. Walmsley et al.^116^ built phototransistors with rise/fall times of about 156/163 μs, which are more than two orders of magnitude faster than other noble‐metal dichalcogenide based phototransistors.

### 4.4 Noble Metal Phosphochalcogenides

The NMPCs have not yet been studied as 2D materials and with this Minireview we want to encourage more research in this direction. Similar to the 2*O* phases,^39^ the NMPCs can be expected to be interesting for photocatalytic applications. Jing et al.^60^ predict few‐layer PdPX to absorb in the visible range and to have matching band edges for the water splitting reaction. This was confirmed by Jiao et al.,^117^ and also extended to the layered Pd_3_(PS_4_)_2_.^118^ The band structures of a single layer of PdPX are more complicated as it consists of two sheets similar to the 2*O* structure, which are connected by an inversion center and P−P bonds (Figure 4 c, Figures S19 and S20). The direct and indirect band gap transitions lie very close in energy, with bulk PdPS being an indirect‐gap semiconductor and bulk PdPSe a direct‐gap semiconductor. As a consequence of the higher structural complexity, they feature many more visible Raman modes (Figure 4 b, Figure S21, and Table S1).

## Biographical Information

*Roman Kempt graduated with an M.Sc. in Chemistry from Leipzig University in 2019. Currently*, *he is a PhD student in the group of Thomas Heine. He investigates novel two‐dimensional materials, such as the noble‐metal dichalcogenides and phosphochalcogenides, with a view to their application in mechanical and optical sensors*.

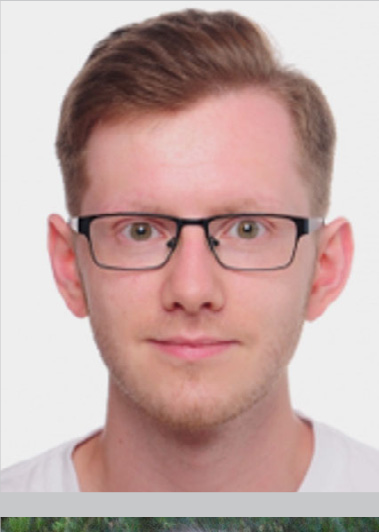

## Biographical Information

*Agnieszka Kuc completed her PhD in physical chemistry at the Technical University of Dresden in the group of Gotthard Seifert in 2008. She obtained her habilitation in physics at Jacobs University in Bremen in the group of Thomas Heine in 2018. She investigates the electronic properties of low‐dimensional materials, including transition‐metal dichalcogenides and oxides, carbon‐based nanostructures, and perovskites using first‐principles methods*.

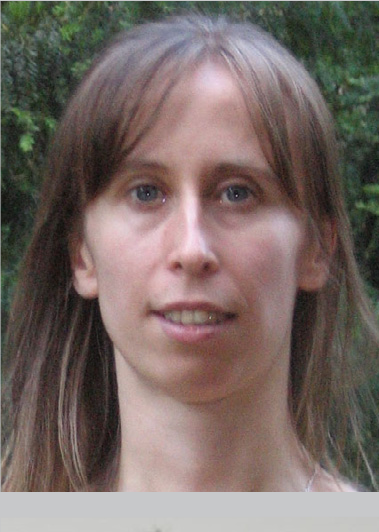

## Biographical Information

*Thomas Heine obtained his PhD at TU Dresden in the group of Gotthard Seifert in 1999. After his habilitation in physical chemistry in 2006, he became professor of theoretical physics and material science at Jacobs University Bremen in 2008. In 2014 he moved to Leipzig University and since 2018 he has been the chair of Theoretical Chemistry at TU Dresden. His group focuses on theoretical materials science*.

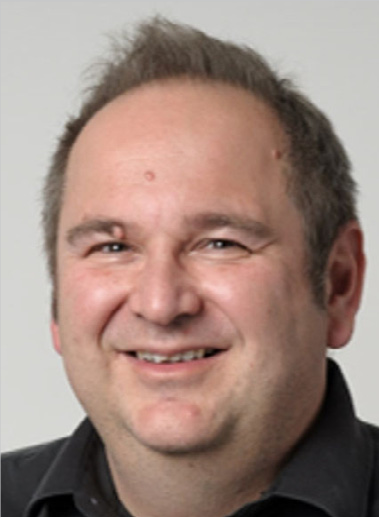

## Supporting information

As a service to our authors and readers, this journal provides supporting information supplied by the authors. Such materials are peer reviewed and may be re‐organized for online delivery, but are not copy‐edited or typeset. Technical support issues arising from supporting information (other than missing files) should be addressed to the authors.

Supplementary
