# Supplementary material for: Two‐Dimensional Noble‐Metal Chalcogenides and Phosphochalcogenides
Source: Angew Chem Int Ed Engl. 2020 Apr 1;59(24):9242–54. doi: 10.1002/anie.201914886 (PMC7463173; doi:10.1002/anie.201914886)
Supplement: Supplementary file 1 — Supplementary [file ANIE-59-9242-s001.pdf]

Angewandte Chemie
Eine Zeitschrift der Gesellschaft Deutscher Chemiker

Supporting Information

Two-Dimensional Noble-Metal Chalcogenides and Phosphochalcogenides**

*Roman Kempt, Agnieszka Kuc, and Thomas Heine**

anie_201914886_sm_miscellaneous_information.pdf

Contents

1 Charge Analysis **3**

2 Computational Method **16**

3 Literature Data and Comparison to this Study **17**
3.1 Experimental Structure Parameters . . . 17
3.2 Calculated Structure Parameters . . . 18

List of Figures

1 Hirshfeld-I charges at the PBE0 level of theory for the bulk compounds in three polytypes. . . . 3
2 Band structures and densities of states (DOS in arbitrary units) of PdS_2 in the $2O$ type for different layer numbers. . . . 4
3 Band structures and densities of states (DOS in arbitrary units) of $PdSe_2$ in the $2O$ type for different layer numbers. . . . 4
4 Band structures and densities of states (DOS in arbitrary units) of high pressure phases of PdS_2 and $PdSe_2$. . . . 4
5 Band structures and densities of states (DOS in arbitrary units) of PtS_2 in the $1T$ type for different layer numbers. . . . 5
6 Band structures and densities of states (DOS in arbitrary units) of $PtSe_2$ in the $1T$ type for different layer numbers. . . . 5
7 Band structures and densities of states (DOS in arbitrary units) of $PtTe_2$ in the $1T$ type for different layer numbers. . . . 5
8 Band structures and densities of states (DOS in arbitrary units) of $PtSe_2$ in the $2H$ type for different layer numbers. . . . 6
9 Band structures and densities of states (DOS in arbitrary units) of $PtSe_2$ in the $2H$ type for different layer numbers. . . . 6
10 Calculated Raman spectra of PtX_2 in the $1T$-type and $2H$-type. . . . 7
11 Band structures and densities of states (DOS in arbitrary units) of $PdTe_2$ in the $1T$ type for different layer numbers. . . . 8
12 Band structures and densities of states (DOS in arbitrary units) of $PdTe_2$, which is stable in the $2O$ type for fewer layer numbers and as pyrite-phase in the bulk. 8
13 Illustration of the $2O$ bulk Raman modes with A_g symmetry. . . . 9
14 Illustration of the $2O$ bulk Raman modes with B_{1g} symmetry. . . . 10
15 Calculated Raman spectra of PdX_2 in the $2O$-type and $1T$-type. . . . 11
16 Calculated IR spectra of NMDCs in various polytypes. . . . 12
17 Band structures and densities of states (DOS in arbitrary units) of PdS_2 in the $1T$ type for different layer numbers. . . . 13
18 Band structures and densities of states (DOS in arbitrary units) of $PdSe_2$ in the $1T$ type for different layer numbers. . . . 13
19 Band structures and densities of states (DOS in arbitrary units) of PdPS for different layer numbers. . . . 13
20 Band structures and densities of states (DOS in arbitrary units) of PdPSe for different layer numbers. . . . 14
21 Calculated Raman spectra of PdPS and PdPSe for different layer numbers. . . . 14

List of Tables

1 Calculated Raman frequencies of bulk PdPS and PdPSe. . . . 15
2 Experimental data on bulk NMDCs and phosphides. . . . 17
3 Comparison of calculated and experimental lattice parameters for different methods from literature and this work ($*$) for PdS_2. . . . 18

4 Comparison of calculated and experimental lattice parameters for different methods from literature and this work (∗) for $PdSe_2$. 18
5 Comparison of calculated and experimental lattice parameters for different methods from literature and this work (∗) for $PdTe_2$. 19
6 Comparison of calculated and experimental lattice parameters for different methods from literature and this work (∗) for PdPS, PdP_2, and PtX_2. 19

1 Charge Analysis

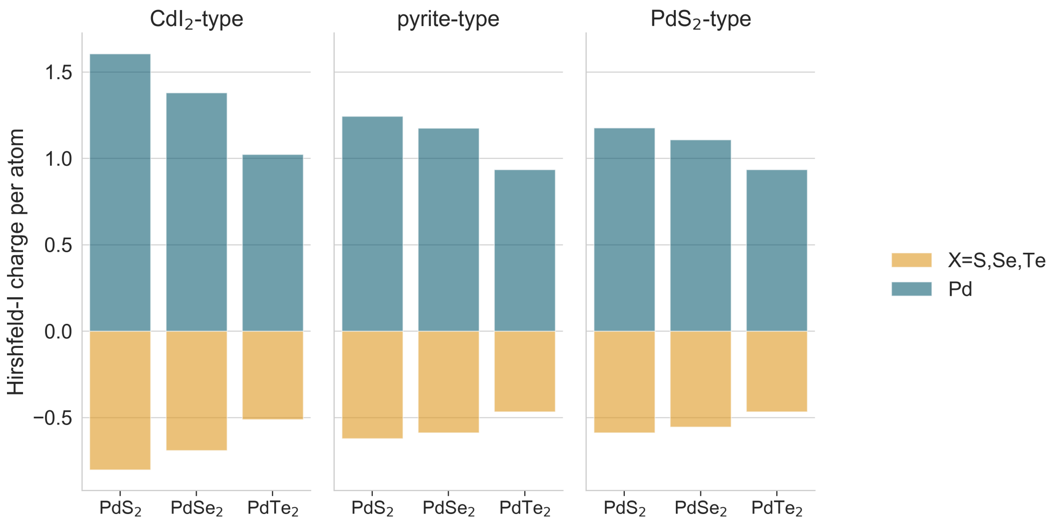

Figure 1: Hirshfeld-I charges at the PBE0 level of theory for the bulk compounds in three polytypes.

The Hirshfeld charge analysis shows a large charge transfer (corresponding to more ionic bonding) in the 1T structures of PdS_2 and $PdSe_2$ in comparison to their pyrite-type structure and the derived PdS_2-type structure. This can be interpreted as a higher oxidation state in the 1T phase. In order to lower their oxidation state, the chalcogens undergo dimer formation, which leads to the pyrite-type and PdS_2-type structures. In the case of $PdTe_2$, the charge transfer between the 1T and pyrite structures has similar magnitude, corresponding to no change in the oxidation state. Since Te-Te dimer formation is less favorable, $PdTe_2$ stays in the 1T structure.

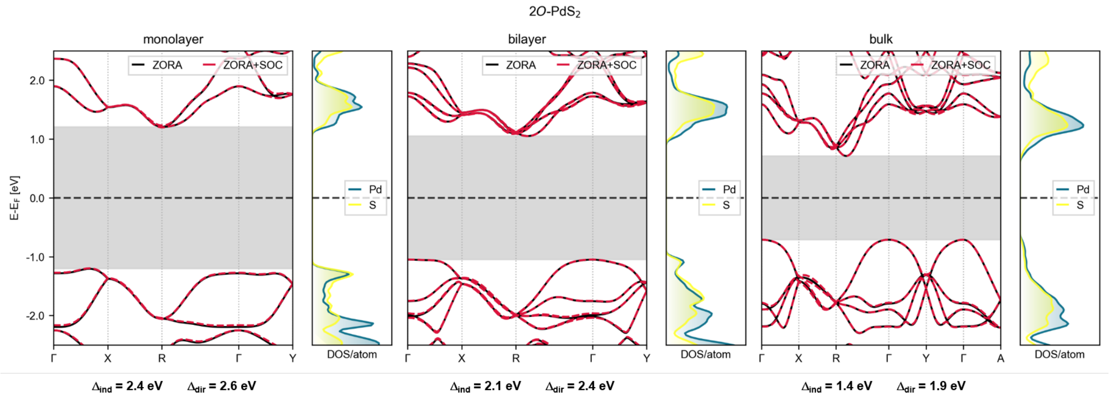

Figure 2: Band structures and densities of states (DOS in arbitrary units) of PdS_2 in the $2O$ type for different layer numbers.

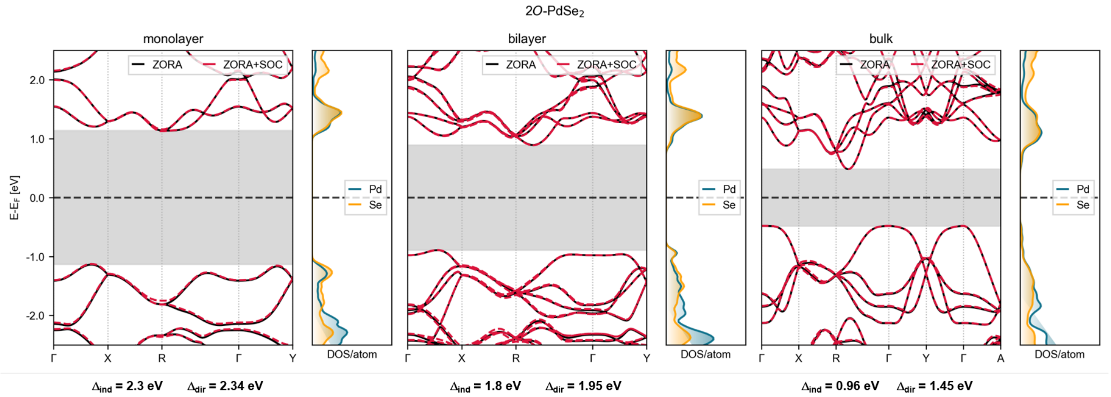

Figure 3: Band structures and densities of states (DOS in arbitrary units) of $PdSe_2$ in the $2O$ type for different layer numbers.

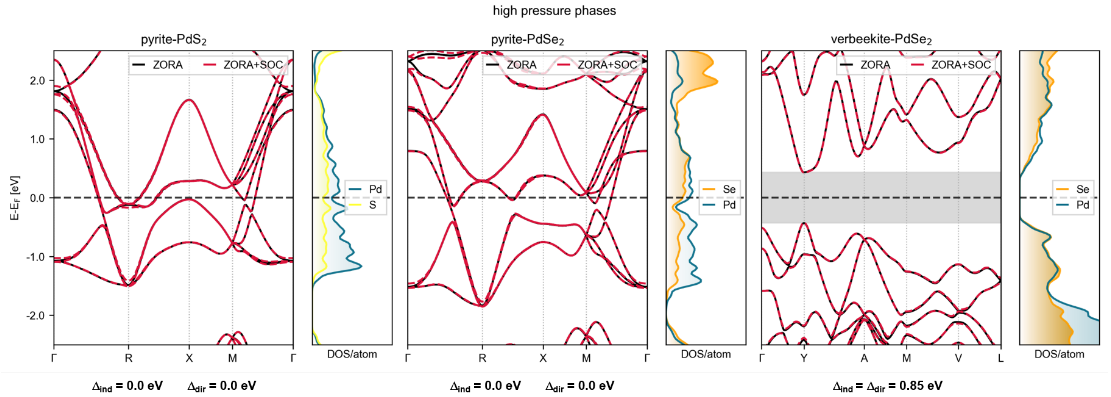

Figure 4: Band structures and densities of states (DOS in arbitrary units) of high pressure phases of PdS_2 and $PdSe_2$.

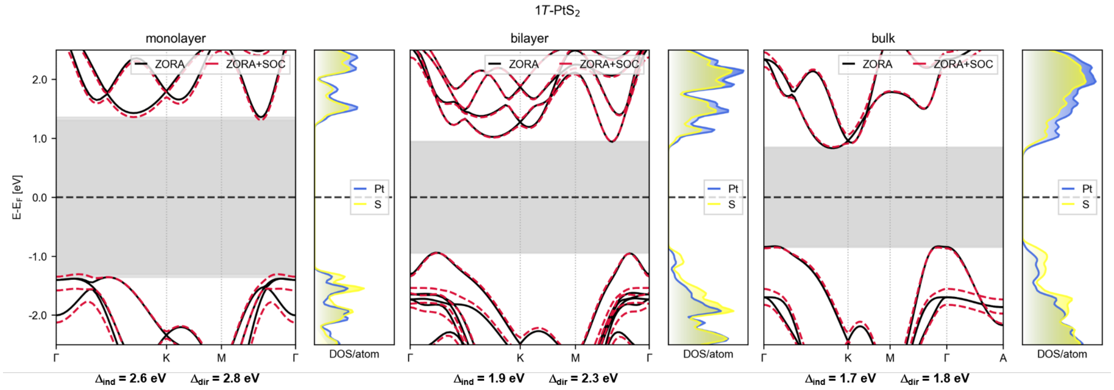

Figure 5: Band structures and densities of states (DOS in arbitrary units) of PtS_2 in the $1T$ type for different layer numbers.

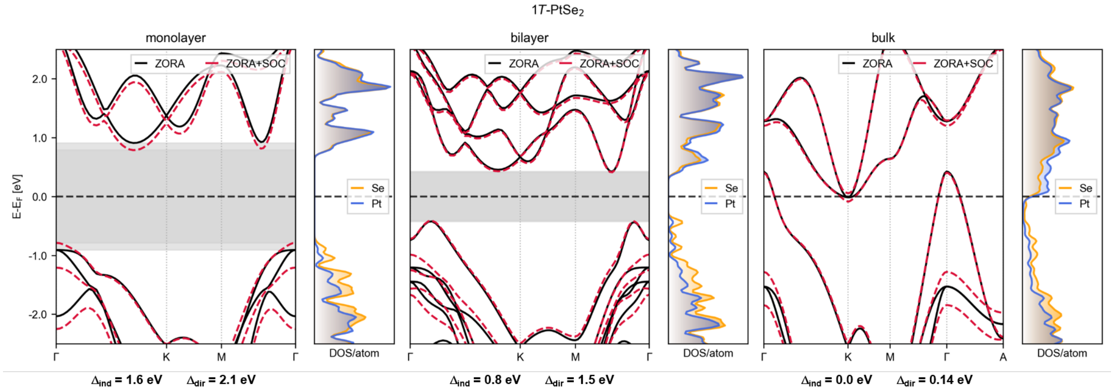

Figure 6: Band structures and densities of states (DOS in arbitrary units) of $PtSe_2$ in the $1T$ type for different layer numbers.

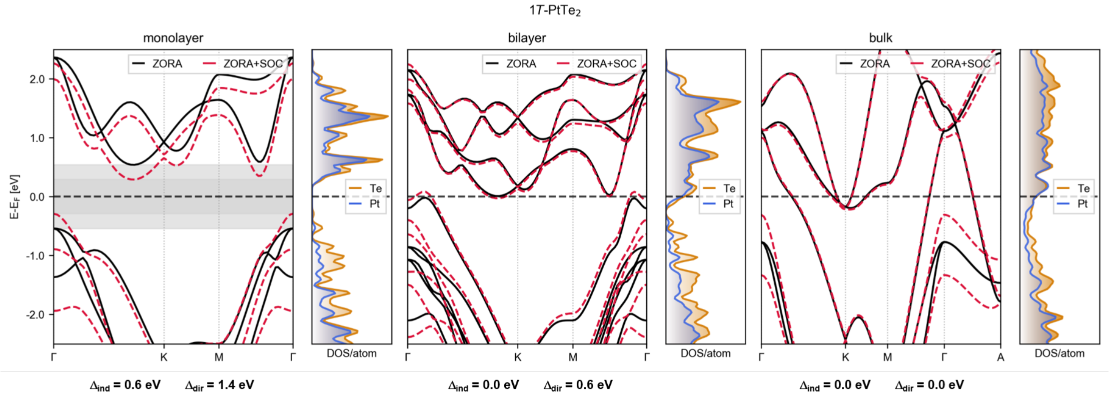

Figure 7: Band structures and densities of states (DOS in arbitrary units) of $PtTe_2$ in the $1T$ type for different layer numbers.

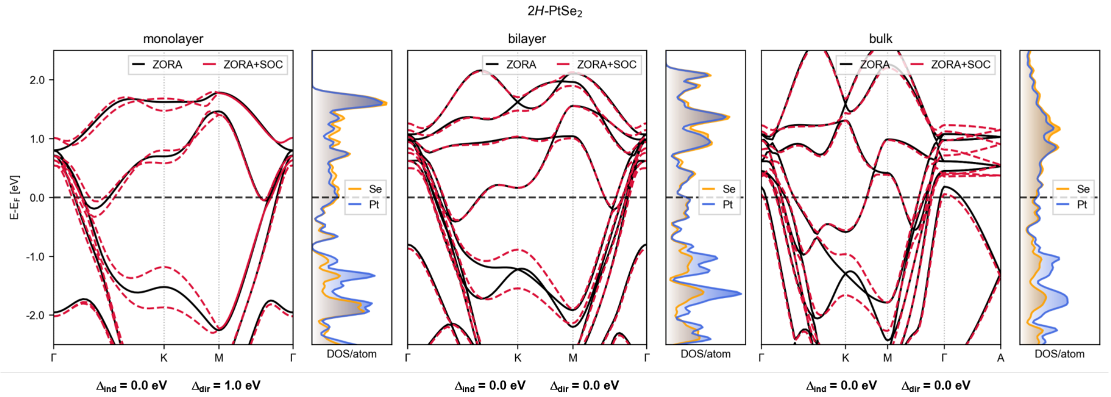

Figure 8: Band structures and densities of states (DOS in arbitrary units) of $PtSe_2$ in the $2H$ type for different layer numbers.

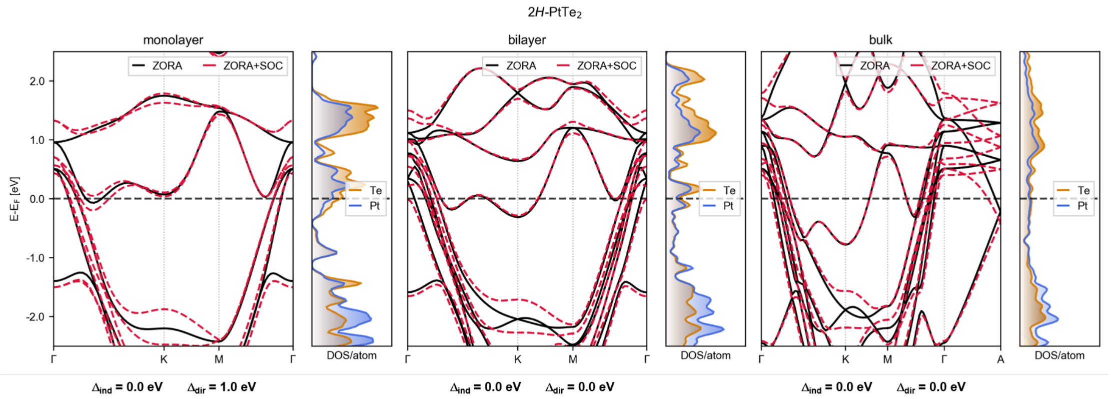

Figure 9: Band structures and densities of states (DOS in arbitrary units) of $PtSe_2$ in the $2H$ type for different layer numbers.

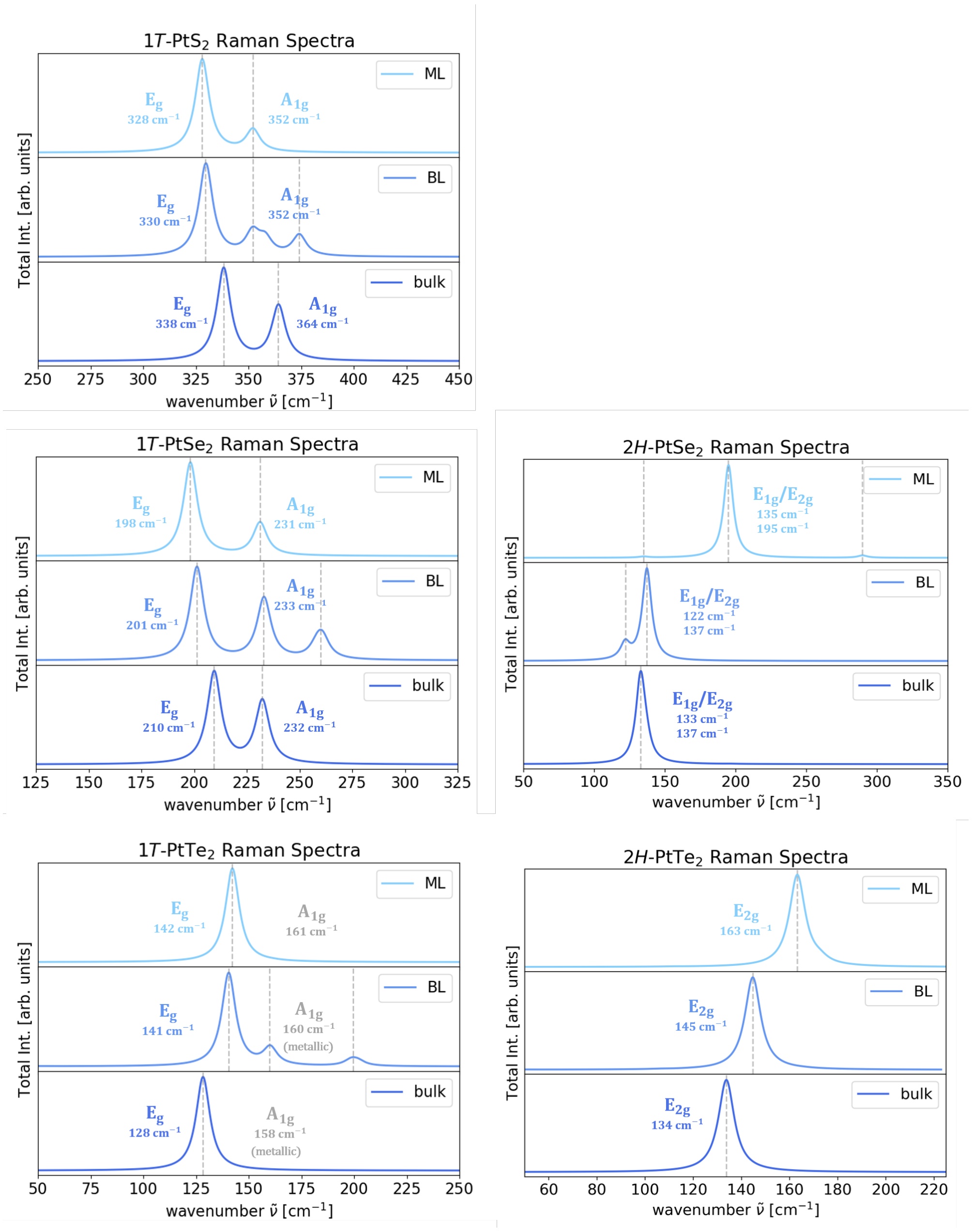

Figure 10: Calculated Raman spectra of PtX_2 in the $1T$-type and $2H$-type.

We consider $2H$-PtS_2 to be dynamically instable within our range of investigated structures due to the appearence of imaginary frequencies. This suggests that $2H$-PtS_2 is instable towards its own vibrations, but does not exclude that it can occur as a metastable phase.

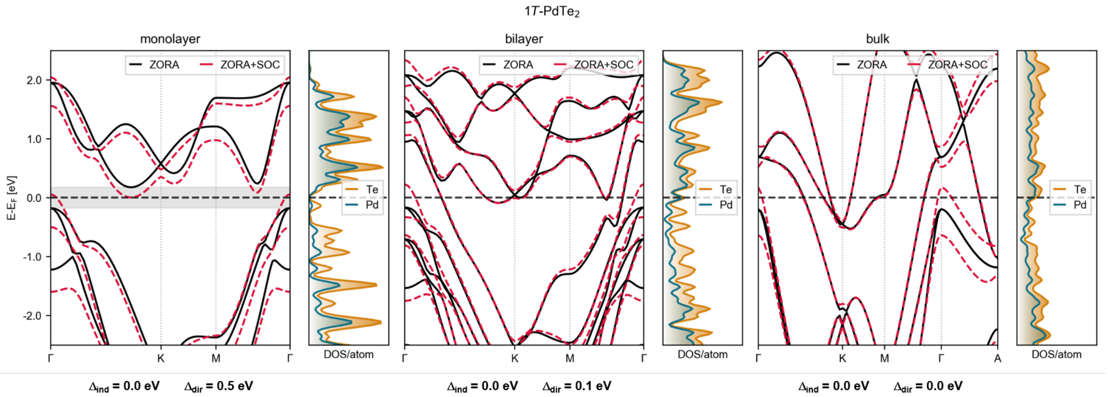

Figure 11: Band structures and densities of states (DOS in arbitrary units) of $PdTe_2$ in the $1T$ type for different layer numbers.

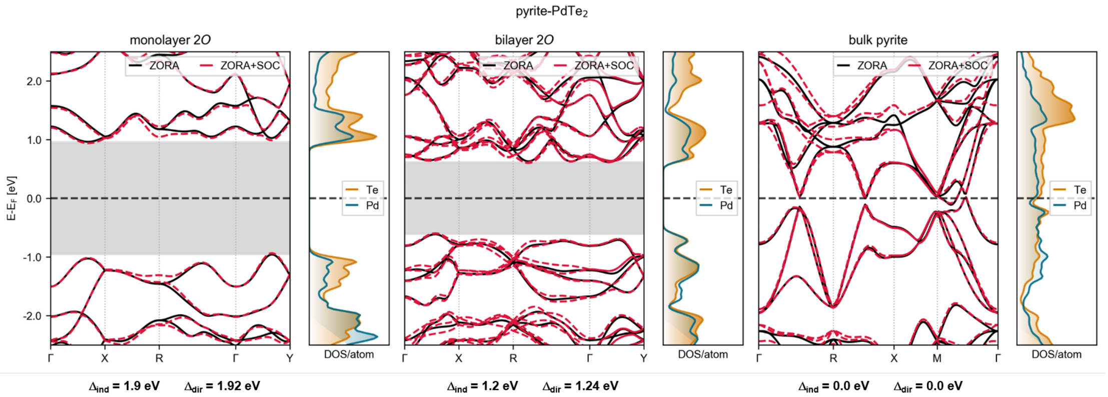

Figure 12: Band structures and densities of states (DOS in arbitrary units) of $PdTe_2$, which is stable in the $2O$ type for fewer layer numbers and as pyrite-phase in the bulk.

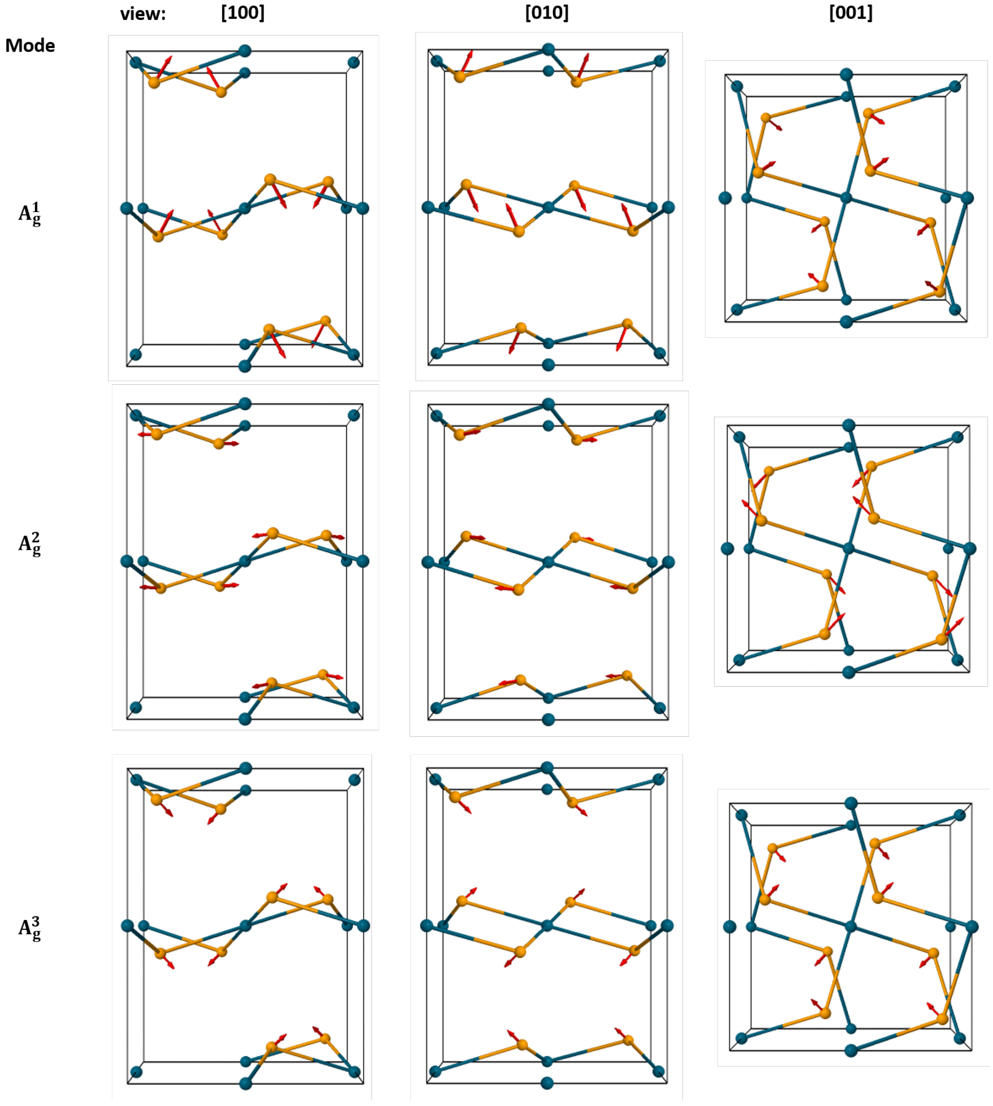

Figure 13: Illustration of the 2O bulk Raman modes with A_g symmetry.

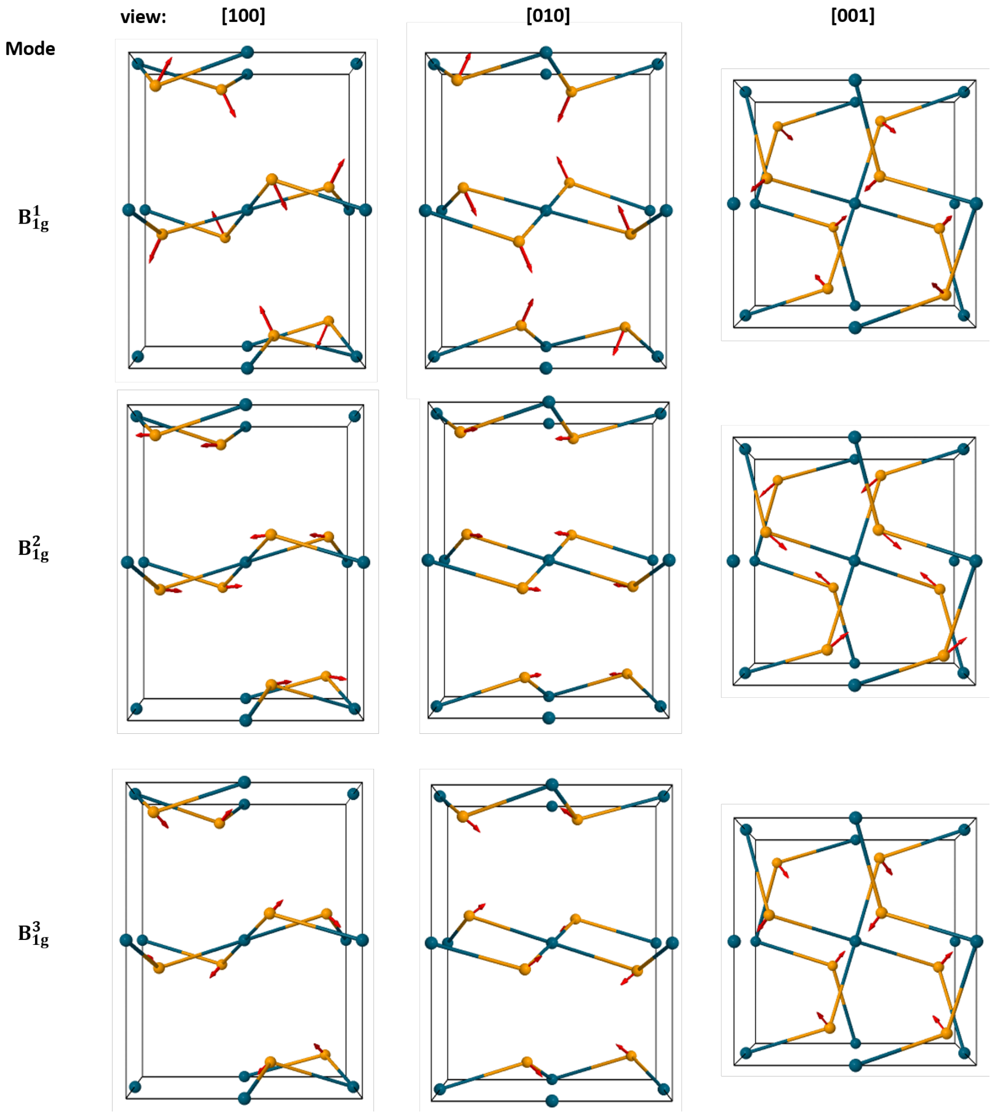

Figure 14: Illustration of the 2O bulk Raman modes with B_{1g} symmetry.

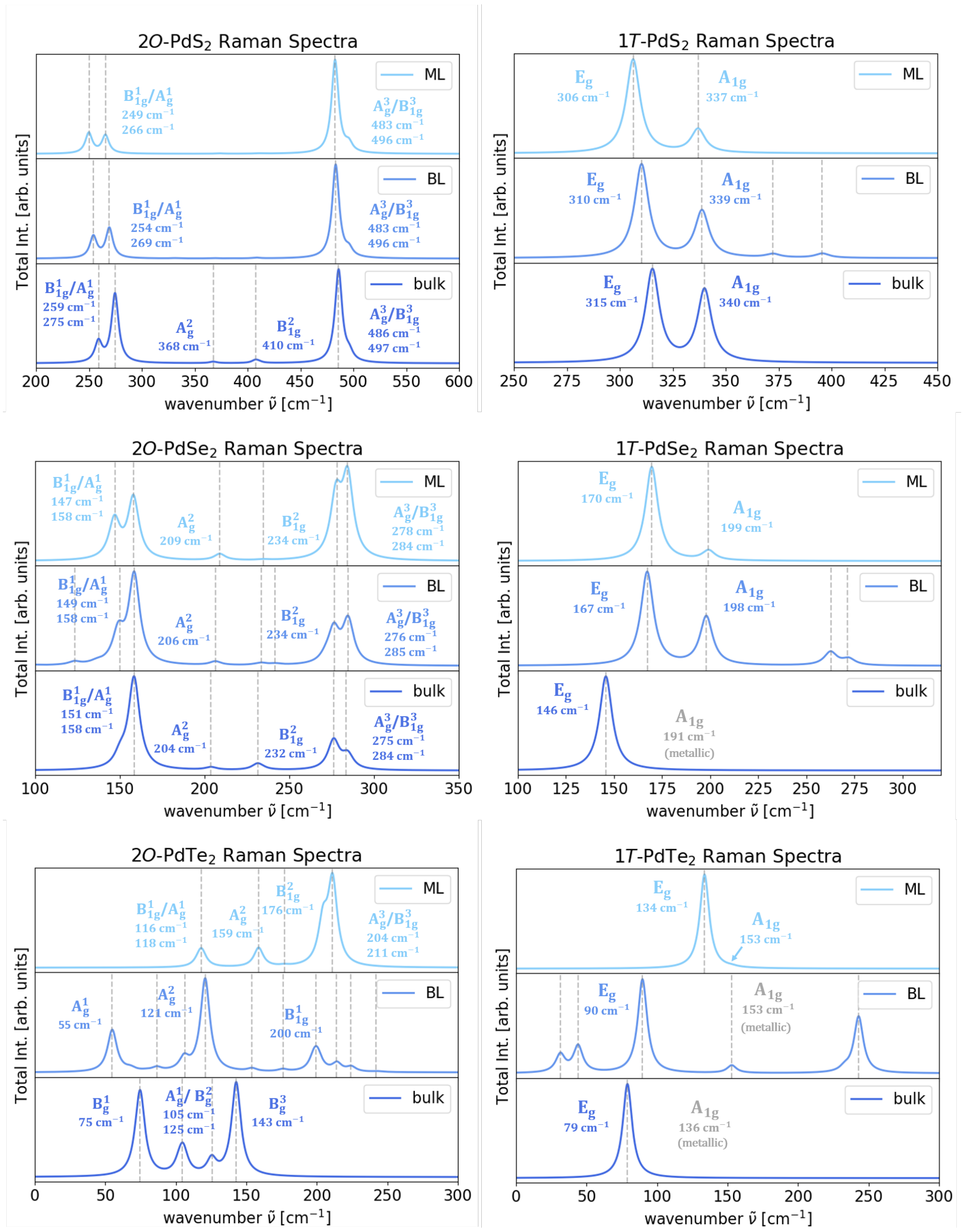

Figure 15: Calculated Raman spectra of PdX_2 in the $2O$-type and $1T$-type.

Concerning metallic systems, the intensities of Raman modes are difficult to calculate and will require future investigations. Here, we indicate the peak positions in gray.

Bulk $2O$-$PdTe_2$ is identical to the pyrite-type, which has higher symmetry. Hence, the calculated Raman spectrum changes a lot with the layer number.

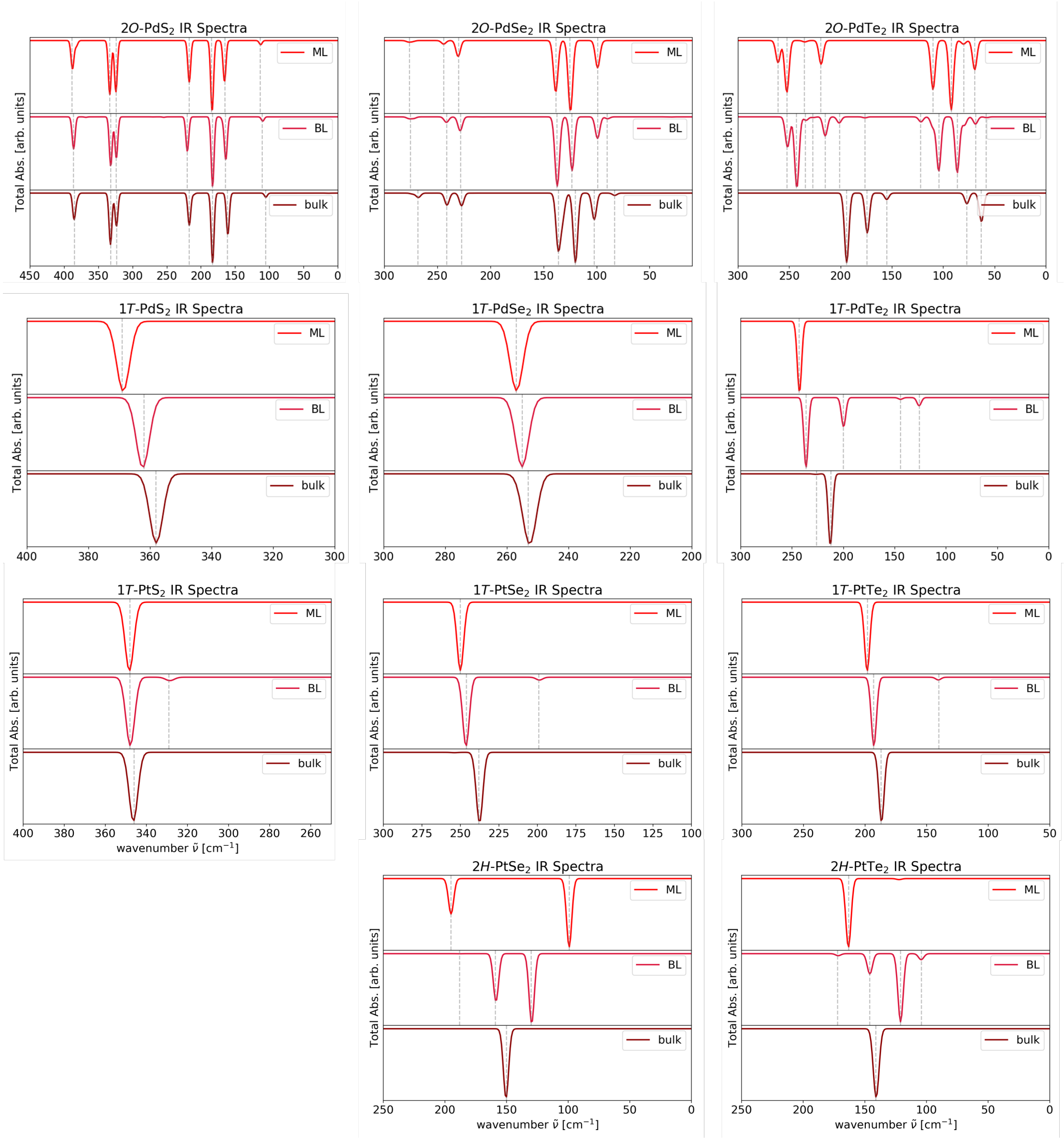

Figure 16: Calculated IR spectra of NMDCs in various polytypes.

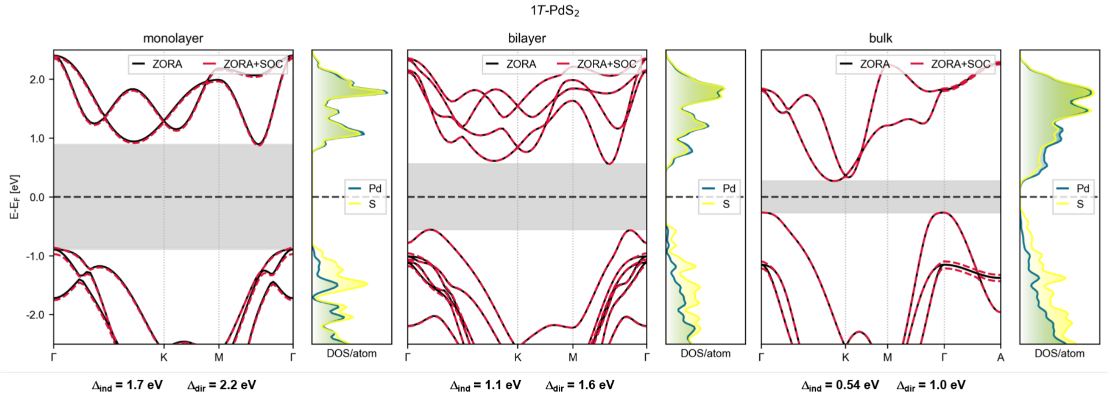

Figure 17: Band structures and densities of states (DOS in arbitrary units) of PdS_2 in the $1T$ type for different layer numbers.

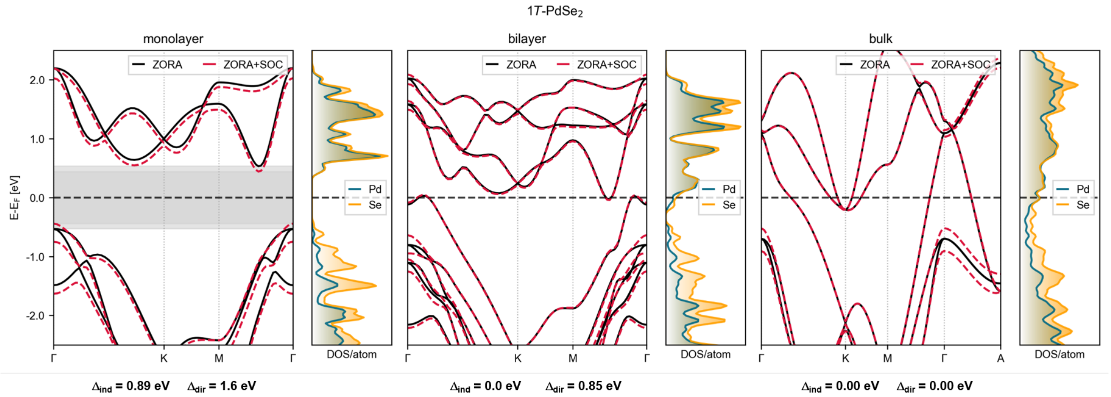

Figure 18: Band structures and densities of states (DOS in arbitrary units) of $PdSe_2$ in the $1T$ type for different layer numbers.

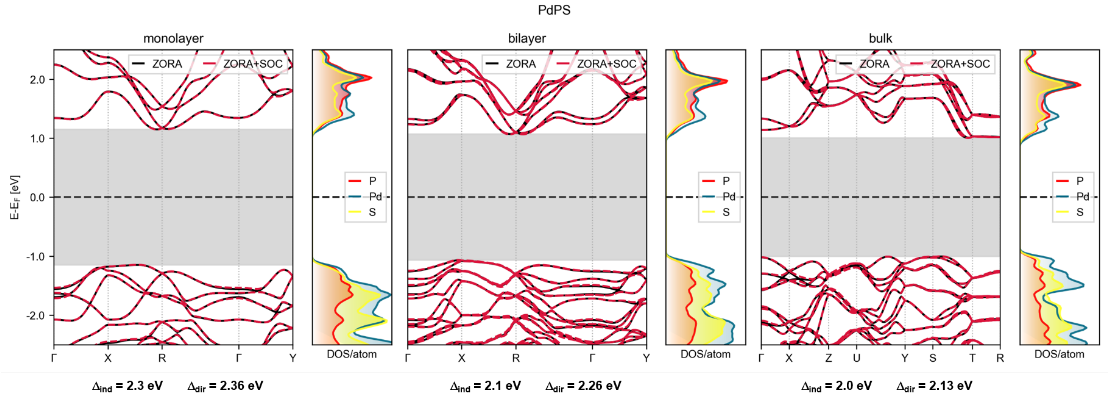

Figure 19: Band structures and densities of states (DOS in arbitrary units) of PdPS for different layer numbers.

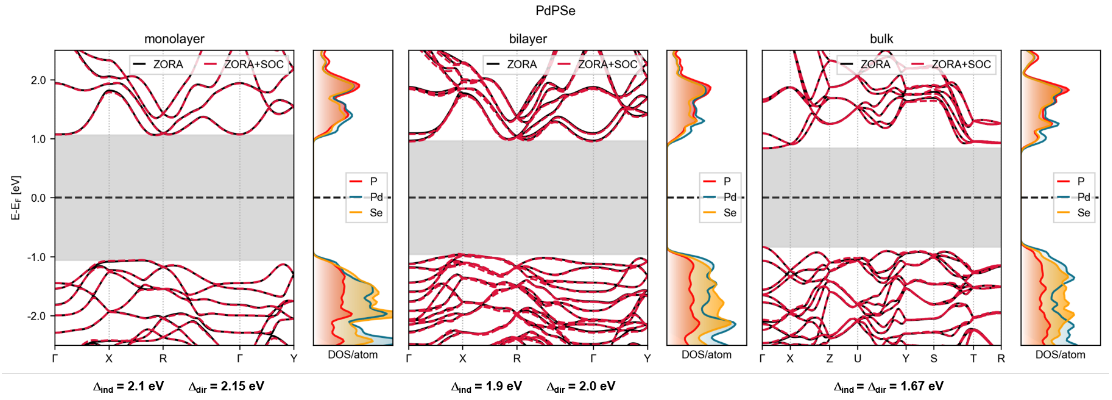

Figure 20: Band structures and densities of states (DOS in arbitrary units) of PdPSe for different layer numbers.

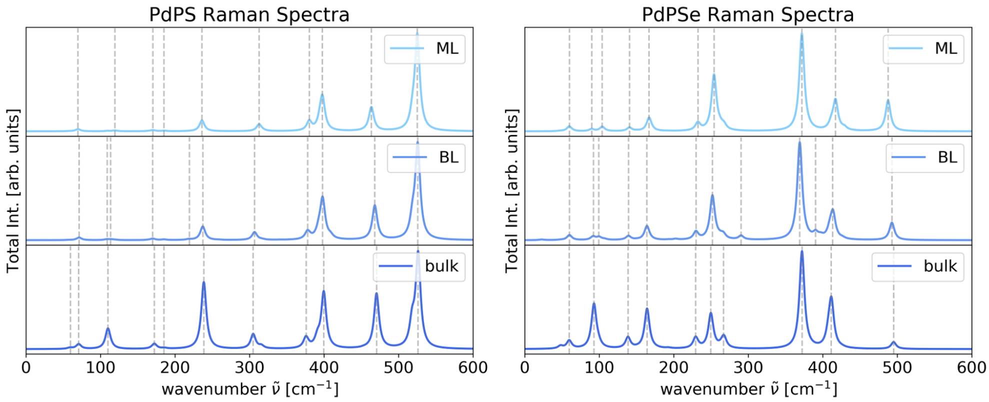

Figure 21: Calculated Raman spectra of PdPS and PdPSe for different layer numbers.

Table 1: Calculated Raman frequencies of bulk PdPS and PdPSe.

bulk PdPS		bulk PdPSe	
Mode	Freq. [$\tilde{\nu}$]	Mode	Freq. [$\tilde{\nu}$]
B_{1g}	59.15	B_{1g}	48.0
A_g	70.73	A_g	59.7
A_g	110.0	A_g	92.9
B_{2g}	112.4	B_{2g}	98.7
A_g	172.3	B_{1g}	98.9
B_{2g}	185.6	B_{3g}	137.6
A_g	238.6	A_g	138.6
B_{2g}	240.3	A_g	164.2
A_g	304.9	A_g	229.2
B_{1g}	315.8	B_{3g}	231.1
A_g	375.6	A_g	249.7
B_{3g}	377.3	B_{3g}	251.0
B_{1g}	390.0	B_{2g}	265.6
B_{2g}	391.3	B_{1g}	267.8
A_g	399.7	A_g	372.1
A_g	470.5	B_{1g}	406.4
B_{2g}	518.3	B_{2g}	407.8
A_g	526.3	A_g	411.6
-	-	A_g	495.1

2 Computational Method

Due to the importance of electron correlation, common DFT approaches, such as PBE-D3 or PBE-MBD, easily misjudge phase stabilities in the case of the Noble-Metal Dichalcogenides (NMDCs). [1–7] The performance of different approaches is summarized in Tables 3, 4, 5 and 6. To obtain reliable predictions, we employ the PBE0 functional [8] to calculate all structural features, such as stabilities, ground-state structures, and vibrational spectra. We show that this approach yields mean average deviations of ≈5 % from experimental structures.

In detail, we performed full relaxation of lattice vectors and atomic positions with Crystal17 [9] and FHI-AIMS [10] (the latter for calculations including many-body-dispersion). We started from experimental structures where available (see Table 2). Hypothetical structures were varied with respect to their lattice parameters and subsequently re-optimised. Calculations in Crystal17 were generally performed on a triple-ζ valence polarisation (TZVP) basis. [11] In case of Pt, only a double-ζ valence polarisation basis was available. [12] In FHI-AIMS, the *tight* tier 2 basis sets were used. All relaxations were performed on sufficiently large k-grids of at least $18\times18(\times18)$ k-points, where for metallic systems the sampling was increased up to $24\times24(\times24)$. In Crystal17, the structures were optimised till the root-mean-square deviations of the gradients were smaller than 0.00003 a.u. and of the estimated displacements smaller than 0.00012 a.u., while the energy change between subsequent SCF cycles was optimised till below 10^{-8} a.u. In case of metallic systems, the Fermi-surface was smeared by a finite temperature of 0.02 a.u. In FHI-AIMS, all forces were relaxed till below 5×10^{-3} eV/Å. All optimised structures showed non-negative Γ-point frequencies.

For optimisation, we investigated the GGA-type functional by Perdew, Burke and Ernzerhof (PBE) [13] including different corrections for the dispersion energy, such as DFT-D by Grimme et al. [14–16], Becke-Johnson damping (BJ) [17], and the many-body-dispersion correction (MBD). [18, 19] Furthermore, we employed the hybrid functional PBE0 [8] and the range-separated hybrid functional from Heyd, Scuseria and Ernzerhof HSE06 [20–22]. The hybrid functionals were the only functionals that correctly reproduced experimental phase stabilities in our investigations.

Electronic structure calculations, including band structures and densities of states, were carried out in FHI-AIMS including full treatment of relativistic effects through the Zeroth Order Regular Approximation (ZORA) and Spin-Orbit Coupling (SOC) with the HSE06 functional. [22] Raman and IR spectra were predicted employing the Coupled Perturbed Kohn-Sham approach [23–25] on optimised structures as implemented in Crystal17 [9] with PBE0.

3 Literature Data and Comparison to this Study

3.1 Experimental Structure Parameters

Table 2: Experimental data on bulk NMDCs and phosphides.

System	Type	a	b	c	α	β	γ	V	Δ	Ref.
PdS_2	PdS_2	5.460	5.416	7.531	90	90	90	228	0.70	[26, 27]
PdS_2	PdS_2-II	5.520	5.551	7.202	90	90	90	221	-	[28]
$PdSe_2$	PdS_2	5.741	5.866	7.691	90	90	90	259	0.50	[26, 27, 29]
$PdSe_2$	Marcasite	4.873	6.013	3.930	90	90	90	115	-	[30]
$PdSe_2$	Pyrite	6.100	6.100	6.100	90	90	90	227	0.0	[30]
$PdSe_2$	Verbeekite	10.93	4.154	6.710	90	125	90	248	-	[31]
$PdTe_2$	CdI_2	4.037	4.037	5.126	90	90	120	72.3	0.0	[27, 32]
PdP_2	PdP_2	6.779	5.857	5.874	90	122	90	99.1	-	[33]
PdPS	PdPS	13.31	5.678	5.693	90	90	90	430	0.65	[34, 35]
PdPSe	PdPS	13.57	5.824	5.856	90	90	90	463	0.15	[34, 35]
PdSSe	PdS_2	5.595	5.713	7.672	90	90	90	245	0.70	[27]
PdSeTe	CdI_2	3.900	3.900	4.980	90	90	120	65.6	0.0	[27]
PtS_2	CdI_2	3.543	3.543	5.039	90	90	120	54.8	0.70	[27, 36]
$PtSe_2$	CdI_2	3.728	3.728	5.081	90	90	120	61.2	0.0	[36, 37]
$PtTe_2$	CdI_2	4.026	4.026	5.221	90	90	120	71.3	0.0	[27, 36]

Lattice parameters (a, b, c) are given in Angström Å, angles (α, β, γ) in degrees, conventional unit cell volume V in Å^3, and band gaps Δ are given in eV.

3.2 Calculated Structure Parameters

We define the MAD as:

$$MAD = \sqrt{\left(\frac{\Delta a}{a}\right)^2 + \left(\frac{\Delta b}{b}\right)^2 + \left(\frac{\Delta c}{c}\right)^2 + \left(\frac{\Delta \alpha}{\alpha}\right)^2 + \left(\frac{\Delta \beta}{\beta}\right)^2 + \left(\frac{\Delta \gamma}{\gamma}\right)^2} \cdot 100\% \qquad (1)$$

Where the reference is the available experimental data.
In the following tables, Cr17 refers to Crystal17, VASP to *Vienna Ab Initio Simulation Package*, and QE to *Quantum Espresso*. PAW represents projector augmented waves, LCAO refers to linear combination of atomic orbitals, and TMNCPP to Troullier-Martins type norm-conserving pseudopotentials. For details on the method please refer to the reference. MAD is the mean average deviation in %.

Table 3: Comparison of calculated and experimental lattice parameters for different methods from literature and this work (∗) for PdS_2.

System	Structure	a	b	c	α	β	γ	MAD	Method	Ref.
PdS_2	PdS_2	5.465	5.538	7.525	90	90	90	2.26	LDA+PW/PAW(VASP)	[38]
PdS_2	PdS_2	5.485	5.582	7.661	90	90	90	3.55	PBE/LCAO(Cr17)	∗
PdS_2	PdS_2	5.475	5.578	7.697	90	90	90	3.73	PBE-D2/LCAO(Cr17)	∗
PdS_2	PdS_2	5.851	5.841	5.840	90	90	90	24.84	PBE-D3/LCAO(Cr17)	∗
PdS_2	PdS_2	5.829	5.836	5.822	90	90	90	24.92	PBE-D3(BJ)/LCAO(Cr17)	∗
PdS_2	PdS_2	5.644	5.694	6.773	90	90	90	11.79	PBE-MBD/LCAO(AIMS)	∗
PdS_2	PdS_2	5.429	5.527	7.894	90	90	90	5.27	HSE06/LCAO(Cr17)	∗
PdS_2	PdS_2	5.424	5.494	7.083	90	90	90	6.16	HSE06-D3(BJ)/LCAO(Cr17)	∗
PdS_2	PdS_2	5.416	5.521	7.946	90	90	90	5.9	PBE0/LCAO(Cr17)	∗
PdS_2	CdI_2	3.483	3.483	5.226	90	90	120	-	PBE0/LCAO(Cr17)	∗
PdS_2	Pyrite	5.855	5.855	5.855	90	90	90	-	PBE0/LCAO(Cr17)	∗
PdS_2	Marcasite	4.793	5.599	3.870	90	90	90	-	PBE0/LCAO(Cr17)	∗

Table 4: Comparison of calculated and experimental lattice parameters for different methods from literature and this work (∗) for $PdSe_2$.

System	Structure	a	b	c	α	β	γ	MAD	Method	Ref.
$PdSe_2$	PdS_2	5.875	5.982	7.72	90	90	90	3.08	LDA+PW/PAW(VASP)	[39]
$PdSe_2$	PdS_2	5.866	6.000	7.356	90	90	90	5.38	LDA+PW/PAW(VASP)	[38]
$PdSe_2$	PdS_2	5.773	5.856	7.384	90	90	90	4.03	DFPTLDA-PZ(CASTEP)	[40]
$PdSe_2$	PdS_2	6.110	6.100	6.120	90	90	90	21.78	LDA/PAW(VASP)	[1]
$PdSe_2$	PdS_2	5.790	5.940	8.480	90	90	90	10.37	PBE/PAW(VASP)	[1]
$PdSe_2$	PdS_2	6.200	6.190	6.210	90	90	90	21.57	DFT-D2/PAW(VASP)	[1]
$PdSe_2$	PdS_2	5.900	6.050	8.470	90	90	90	10.96	vdW-DF/PAW(VASP)	[1]
$PdSe_2$	PdS_2	6.010	6.150	8.340	90	90	90	10.8	vdW-DF2/PAW(VASP)	[1]
$PdSe_2$	PdS_2	5.805	5.990	7.950	90	90	90	4.41	optPBE/PAW(VASP)	[1]
$PdSe_2$	PdS_2	6.220	6.220	6.230	90	90	90	21.61	optB86b/PAW(VASP)	[1]
$PdSe_2$	PdS_2	5.850	5.970	7.630	90	90	90	2.72	optB88/PAW(VASP)	[1]
$PdSe_2$	PdS_2	6.090	6.100	6.100	90	90	90	21.93	LDA/PAW(QE)	[1]
$PdSe_2$	PdS_2	5.800	5.950	8.650	90	90	90	12.59	PBE/PAW(QE)	[1]
$PdSe_2$	PdS_2	5.790	5.920	7.700	90	90	90	1.26	DFT-D2/PAW(QE)	[1]
$PdSe_2$	PdS_2	5.890	6.050	8.680	90	90	90	13.49	vdw-DF/PAW(QE)	[1]
$PdSe_2$	PdS_2	6.010	6.160	8.470	90	90	90	12.23	vdw-DF2/PAW(QE)	[1]
$PdSe_2$	PdS_2	6.160	6.170	6.170	90	90	90	21.71	C09-DF/PAW(QE)	[1]
$PdSe_2$	PdS_2	6.180	6.170	6.190	90	90	90	21.59	C09-DF2/PAW(QE)	[1]
$PdSe_2$	PdS_2	5.840	5.950	7.490	90	90	90	3.44	optB86b/PAW(QE)	[1]
$PdSe_2$	PdS_2	5.860	5.990	7.710	90	90	90	2.97	optB88/PAW(QE)	[1]
$PdSe_2$	PdS_2	5.840	5.960	7.530	90	90	90	3.15	revB86b/PAW(QE)	[1]
$PdSe_2$	PdS_2	6.100	6.110	6.110	90	90	90	21.89	LDA/TMNCPP(QE)	[1]
$PdSe_2$	PdS_2	5.790	5.950	8.720	90	90	90	13.48	PBE/TMNCPP(QE)	[1]
$PdSe_2$	PdS_2	5.780	5.920	7.770	90	90	90	1.54	DFT-D2/TMNCPP(QE)	[1]
$PdSe_2$	PdS_2	5.900	6.050	8.640	90	90	90	13.03	vdW-DF/TMNCPP(QE)	[1]
$PdSe_2$	PdS_2	5.790	5.950	8.680	90	90	90	12.97	vdW-DF2/TMNCPP(QE)	[1]
$PdSe_2$	PdS_2	6.210	6.220	6.210	90	90	90	21.77	C09-DF/TMNCPP(QE)	[1]
$PdSe_2$	PdS_2	6.220	6.230	6.220	90	90	90	21.77	C09-DF2/TMNCPP(QE)	[1]
$PdSe_2$	PdS_2	5.790	5.950	8.680	90	90	90	12.97	optB86b/TMNCPP(QE)	[1]

$PdSe_2$	PdS_2	5.790	5.950	8.720	90	90	90	13.48	optB88/TMNCPP(QE)	[1]
$PdSe_2$	PdS_2	5.790	5.950	8.690	90	90	90	13.1	revB86b/TMNCPP(QE)	[1]
$PdSe_2$	PdS_2	5.786	5.936	8.608	90	90	90	12.01	PBE/PAW(VASP)	[7]
$PdSe_2$	PdS_2	5.848	5.947	7.758	90	90	90	2.48	DFT-D(TS)/PAW(VASP)	[7]
$PdSe_2$	Verbeekite	11.111	4.015	6.728	90	125.0	90	3.76	DFT-D(TS)/PAW(VASP)	[7]
$PdSe_2$	Verbeekite	11.059	3.902	6.632	90	124.6	90	6.33	DFT-D3(BJ)/PAW(VASP)	[7]
$PdSe_2$	Verbeekite	11.068	3.952	6.686	90	125.1	90	5.04	DFT-D3/PAW(VASP)	[7]
$PdSe_2$	Verbeekite	11.107	3.893	6.628	90	124.5	90	6.64	DFT-D2/PAW(VASP)	[7]
$PdSe_2$	Verbeekite	11.145	3.938	6.666	90	124.3	90	5.66	optB86b-vDW/PAW(VASP)	[7]
$PdSe_2$	Verbeekite	11.146	4.159	6.781	90	124.6	90	2.33	optPBE-vdW/PAW(VASP)	[7]
$PdSe_2$	Verbeekite	11.213	4.203	6.821	90	124.7	90	3.34	optB88-vdW/PAW(VASP)	[7]
$PdSe_2$	Verbeekite	11.468	3.751	6.500	90	124.5	90	11.35	PBEsol/PAW(VASP)	[7]
$PdSe_2$	Verbeekite	11.055	4.153	6.755	90	124.8	90	1.41	PBE/PAW(VASP)	[7]
$PdSe_2$	PdS_2	5.779	5.931	8.495	90	90	90	10.53	PBE/PAW(VASP)	[2]
$PdSe_2$	PdS_2	6.196	6.196	6.193	90	90	90	21.77	PBE-D2/PAW(VASP)	[2]
$PdSe_2$	PdS_2	6.198	6.197	6.192	90	90	90	21.8	PBE-D3(ZP)/PAW(VASP)	[2]
$PdSe_2$	PdS_2	6.179	6.178	6.173	90	90	90	21.82	PBE-D3(BJ)/PAW(VASP)	[2]
$PdSe_2$	PdS_2	6.208	6.210	6.201	90	90	90	21.81	PBE-dDsC/PAW(VASP)	[2]
$PdSe_2$	PdS_2	6.310	6.310	6.303	90	90	90	21.94	optPBE/PAW(VASP)	[2]
$PdSe_2$	PdS_2	6.220	6.221	6.212	90	90	90	21.82	optB86b/PAW(VASP)	[2]
$PdSe_2$	PdS_2	6.268	6.269	6.258	90	90	90	21.88	optB88/PAW(VASP)	[2]
$PdSe_2$	PdS_2	6.512	6.513	6.524	90	90	90	23.07	vdW-DF2/PAW(VASP)	[2]
$PdSe_2$	PdS_2	5.862	5.953	7.59	90	90	90	2.89	PBE-TS/PAW(VASP)	[2]
$PdSe_2$	PdS_2	5.859	5.955	7.591	90	90	90	2.87	PBE-TS(HI)/PAW(VASP)	[2]
$PdSe_2$	PdS_2	5.851	5.947	7.694	90	90	90	2.36	PBE-TS(SCS)/PAW(VASP)	[2]
$PdSe_2$	PdS_2	6.058	6.082	6.688	90	90	90	14.63	PBE/LCAO(Cr17)	*
$PdSe_2$	PdS_2	6.017	6.057	6.825	90	90	90	12.67	PBE-D2/LCAO(Cr17)	*
$PdSe_2$	PdS_2	6.183	6.173	6.19	90	90	90	21.62	PBE-D3/LCAO(Cr7)	*
$PdSe_2$	PdS_2	6.171	6.159	6.166	90	90	90	21.78	PBE-D3(BJ)/LCAO(Cr17)	*
$PdSe_2$	PdS_2	6.234	6.234	6.234	90	90	90	21.73	PBE-MBD/LCAO(AIMS)	*
$PdSe_2$	PdS_2	5.734	5.874	7.998	90	90	90	4.0	HSE06/LCAO(Cr17)	*
$PdSe_2$	PdS_2	5.722	5.830	7.304	90	90	90	5.08	HSE06-D3(BJ)/LCAO(Cr17)	*
$PdSe_2$	PdS_2	5.718	5.864	8.048	90	90	90	4.66	PBE0/LCAO(Cr17)	*
$PdSe_2$	Pyrite	6.194	6.194	6.194	90	90	90	2.67	PBE0/LCAO(Cr17)	*
$PdSe_2$	Marcasite	5.135	5.938	4.06	90	90	90	6.43	PBE0/LCAO(Cr17)	*
$PdSe_2$	Verbeekite	10.850	4.154	6.689	90	125.6	90	0.81	PBE0/LCAO(Cr17)	*
$PdSe_2$	CdI_2	3.734	3.734	4.889	90	90	120	-	PBE0/LCAO(Cr17)	*

Table 5: Comparison of calculated and experimental lattice parameters for different methods from literature and this work (*) for $PdTe_2$.

System	Structure	a	b	c	α	β	γ	MAD	Method	Ref.
$PdTe_2$	CdI_2	4.097	4.097	5.19	90	90	120	2.44	PBE/PAW(Vasp)	[41]
$PdTe_2$	CdI_2	4.033	4.033	5.231	90	90	120	2.05	PBE/LCAO(Cr17)	*
$PdTe_2$	CdI_2	4.023	4.023	5.206	90	90	120	1.64	PBE-D2/LCAO(Cr17)	*
$PdTe_2$	CdI_2	4.016	4.016	5.034	90	90	120	1.94	PBE-D3/LCAO(Cr17)	*
$PdTe_2$	CdI_2	3.989	3.989	5.069	90	90	120	2.02	PBE-D3(BJ)/LCAO(Cr17)	*
$PdTe_2$	CdI_2	4.077	4.077	5.147	90	90	120	1.46	PBE-MBD/LCAO(AIMS)	*
$PdTe_2$	CdI_2	4.009	4.009	5.184	90	90	120	1.5	HSE06/LCAO(Cr17)	*
$PdTe_2$	CdI_2	3.963	3.963	4.972	90	90	120	3.97	HSE06-D3(BJ)/LCAO(Cr17)	*
$PdTe_2$	CdI_2	4.006	4.006	5.106	90	90	120	1.15	PBE0/LCAO(Cr17)	*
$PdTe_2$	Pyrite	6.561	6.561	6.561	90	90	90	-	PBE0/LCAO(Cr17)	*

Table 6: Comparison of calculated and experimental lattice parameters for different methods from literature and this work (*) for PdPS, PdP_2, and PtX_2.

System	Structure	a	b	c	α	β	γ	MAD	Method	Ref.
PdPS	PdPS	13.607	5.674	5.687	90	90	90	2.238	PBE0/LCAO(Cr17)	*
PdPSe	PdPS	13.900	5.831	5.866	90	90	90	2.403	PBE0/LCAO(Cr17)	*

PdP_2	PdP_2	6.785	5.850	5.887	90	121.9	90	0.3	LDA+PW/PAW(VASP)	[38]
PdP_2	PdP_2	6.815	5.867	6.239	90	126.7	90	7.44	PBE0/LCAO(Cr17)	*
PtS_2	CdI_2	3.570	3.570	6.320	90	90	120	25.45	PBE/PAW(VASP)	[42]
PtS_2	CdI_2	3.600	3.600	4.600	90	90	120	9.0	PBE+D3/PAW(VASP)	[42]
PtS_2	CdI_2	3.530	3.530	5.317	90	90	120	5.54	PBE0/LCAO(Cr17)	*
$PtSe_2$	CdI_2	3.709	3.709	4.933	90	90	120	3.01	PBE0/LCAO(Cr17)	*
$PtSe_2$	MoS_2	3.507	3.507	11.334	90	90	120	-	PBE0/LCAO(Cr17)	*
$PtSe_2$	CdI_2	3.750	3.750	6.550	90	90	120	28.92	PBE/PAW(VASP)	[42]
$PtSe_2$	CdI_2	3.790	3.790	4.750	90	90	120	6.93	PBE+D3/PAW(VASP)	[42]
$PtTe_2$	CdI_2	4.031	4.031	5.275	90	90	120.0	1.04	PBE0/LCAO(Cr17)	*
$PtTe_2$	MoS_2	3.879	3.879	11.957	90	90	120.0	-	PBE0/LCAO(Cr17)	*

References

1. Oyedele, A. D. *et al.* $PdSe_2$: Pentagonal Two-Dimensional Layers with High Air Stability for Electronics. *Journal of the American Chemical Society* **139,** 14090–14097 (2017).
2. Sun, M. *et al.* Few-Layer $PdSe_2$ Sheets: Promising Thermoelectric Materials Driven by High Valley Convergence. *ACS Omega* **3,** 5971–5979 (2018).
3. Wang, Y., Li, Y. & Chen, Z. Not Your Familiar Two Dimensional Transition Metal Disulfide: Structural and Electronic Properties of the PdS_2 Monolayer. *Journal of Materials Chemistry C* **3,** 9603–9608 (2015).
4. Ghorbani-Asl, M. *et al.* A Single-Material Logical Junction Based on 2D Crystal PdS_2. *Advanced Materials* **28,** 853–856 (2016).
5. Lei, W. *et al.* Ferroelastic Lattice Rotation and Band-Gap Engineering in Quasi 2D Layered-Structure $PdSe_2$ under Uniaxial Stress. *Nanoscale* (2019).
6. Liu, G. *et al.* Negative Poisson's Ratio in Monolayer $PdSe_2$. *Computational Materials Science* **160,** 309–314 (2019).
7. Lei, W. *et al.* A New 2D High-Pressure Phase of $PdSe_2$ with High-Mobility Transport Anisotropy for Photovoltaic Applications. *Journal of Materials Chemistry C* **7,** 2096–2105 (2019).
8. Perdew, J. P., Ernzerhof, M. & Burke, K. Rationale for Mixing Exact Exchange with Density Functional Approximations. *J. Chem. Phys.* **105,** 9982 (1996).
9. Dovesi, R. *et al.* Quantum-Mechanical Condensed Matter Simulations with CRYSTAL. *Wiley Interdisciplinary Reviews: Computational Molecular Science* **8,** e1360 (2018).
10. Blum, V. *et al.* Ab Initio Molecular Simulations with Numeric Atom-Centered Orbitals. *Computer Physics Communications* **180,** 2175–2196 (2009).
11. Laun, J., Vilela Oliveira, D. & Bredow, T. Consistent Gaussian Basis Sets of Double- and Triple-Zeta Valence with Polarization Quality of the Fifth Period for Solid-State Calculations. eng. *Journal of Computational Chemistry* **39,** 1285–1290 (2018).
12. Kokalj, A. *et al.* Periodic DFT Study of the Pt(111): A p(1×1) Atomic Oxygen Interaction with the Surface. *The Journal of Physical Chemistry B* **103,** 7222–7232 (1999).
13. Perdew, J. P., Burke, K. & Ernzerhof, M. Generalized Gradient Approximation Made Simple. *Physical Review Letters* **77,** 3865–3868 (1996).
14. Grimme, S. Semiempirical GGA-type density functional constructed with a long-range dispersion correction. de. *Journal of Computational Chemistry* **27,** 1787–1799 (2006).
15. Grimme, S. *et al.* Dispersion-Corrected Mean-Field Electronic Structure Methods. *Chemical Reviews* **116,** 5105–5154 (2016).
16. Grimme, S. *et al.* A Consistent and Accurate Ab Initio Parametrization of Density Functional Dispersion Correction (DFT-D) for the 94 Elements H-Pu. *The Journal of Chemical Physics* **132,** 154104 (2010).
17. Becke, A. D. & Johnson, E. R. A Simple Effective Potential for Exchange. *The Journal of Chemical Physics* **124,** 221101 (2006).
18. Tkatchenko, A., Ambrosetti, A. & DiStasio, R. A. Interatomic Methods for the Dispersion Energy Derived from the Adiabatic Connection Fluctuation-Dissipation Theorem. *The Journal of Chemical Physics* **138,** 074106 (2013).
19. Tkatchenko, A. *et al.* Accurate and Efficient Method for Many-Body van Der Waals Interactions. *Physical Review Letters* **108,** 236402 (2012).
20. Heyd, J., Scuseria, G. E. & Ernzerhof, M. Hybrid Functionals Based on a Screened Coulomb Potential. *The Journal of Chemical Physics* **118,** 8207–8215 (2003).
21. Heyd, J. & Scuseria, G. E. Efficient Hybrid Density Functional Calculations in Solids: Assessment of the Heyd–Scuseria–Ernzerhof Screened Coulomb Hybrid Functional. *The Journal of Chemical Physics* **121,** 1187–1192 (2004).
22. Heyd, J., Scuseria, G. E. & Ernzerhof, M. Erratum: "Hybrid Functionals Based on a Screened Coulomb Potential" [J. Chem. Phys. 118, 8207 (2003)]. *The Journal of Chemical Physics* **124,** 219906 (2006).
23. Lacivita, V. *et al.* Static and Dynamic Coupled Perturbed Hartree-Fock Vibrational (Hyper)Polarizabilities of Polyacetylene Calculated by the Finite Field Nuclear Relaxation Method. *The Journal of Chemical Physics* **137,** 014103 (2012).

24. Ferrero, M. *et al.* Coupled Perturbed Hartree-Fock for Periodic Systems: The Role of Symmetry and Related Computational Aspects. *The Journal of Chemical Physics* **128,** 014110 (2008).
25. Ferrero, M. *et al.* The Calculation of Static Polarizabilities of 1-3D Periodic Compounds. the Implementation in the Crystal Code. *Journal of Computational Chemistry* **29,** 1450–1459 (2008).
26. Grønvold, F. & Røst, E. The Crystal Structure of $PdSe_2$ and PdS_2. *Acta Crystallographica* **10,** 329–331 (1957).
27. Hulliger, F. Electrical Properties of Some Nickel-Group Chalcogenides. *Journal of Physics and Chemistry of Solids* **26,** 639–645 (1965).
28. Selb, E. *et al.* Crystal Structures of the High-Pressure Palladium Dichalcogenides $Pd_{0.94(1)}S_2$ and $Pd_{0.88(1)}Se_2$ Comprising Exceptional Pd^{IV} Oxidation States. *Zeitschrift für anorganische und allgemeine Chemie* **643,** 1415–1423 (2017).
29. Zhang, G. *et al.* Optical and Electrical Properties of Two-Dimensional Palladium Diselenide. *Applied Physics Letters* **114,** 253102 (2019).
30. Larchev, V. N. & Popova, S. V. Polymorphism of Palladium Dichalcogenides at High Pressures and Temperatures. *Inorganic Materials* **14,** 611–612 (1978).
31. Selb, E., Tribus, M. & Heymann, G. Verbeekite, the Long-Unknown Crystal Structure of Monoclinic $PdSe_2$. *Inorganic Chemistry* **56,** 5885–5891 (2017).
32. Pell, M. A., Mironov, Y. V. & Ibers, J. A. $PdTe_2$. *Acta Crystallographica Section C: Crystal Structure Communications* **52,** 1331–1332 (1996).
33. Zachariasen, W. H. The Crystal Structure of Palladium Diphosphide. *Acta Crystallographica* **16,** 1253–1255 (1963).
34. Jeitschko, W. The Structure of PdPS and the Crystal Chemistry of Late Transition-Metal Dipnictides and Dichalcogenides. *Acta Crystallographica Section B: Structural Crystallography and Crystal Chemistry* **30,** 2565–2572 (1974).
35. Hulliger, F. *Structural Chemistry of Layer-Type Phases* (Springer Netherlands, 1976).
36. Grønvold, F. *et al.* On the Sulfides, Selenides, and Tellurides of Platinum. *Acta Chemica Scandinavica* **14,** 1879–1893 (1960).
37. Wang, Y. *et al.* Monolayer $PtSe_2$, a New Semiconducting Transition-Metal-Dichalcogenide, Epitaxially Grown by Direct Selenization of Pt. *Nano Letters* **15,** 4013–4018 (2015).
38. Hamidani, A., Bennecer, B. & Zanat, K. Structural and Electronic Properties of the Pseudo-Binary Compounds PdX_2 (X=P, S and Se). *Journal of Physics and Chemistry of Solids* **71,** 42–46 (2010).
39. Soulard, C. *et al.* Experimental and Theoretical Investigation on the Relative Stability of the PdS_2- and Pyrite-Type Structures of $PdSe_2$. *Inorganic Chemistry* **43,** 1943–1949 (2004).
40. Chow, W. L. *et al.* High Mobility 2D Palladium Diselenide Field-Effect Transistors with Tunable Ambipolar Characteristics. *Advanced Materials* **29,** 1602969 (2017).
41. Fei, F. *et al.* Nontrivial Berry Phase and Type-II Dirac Transport in the Layered Material $PdTe_2$. *Physical Review B* **96,** 041201 (2017).
42. Piotrowski, M. J., Nomiyama, R. K. & Da Silva, J. L. F. Role of van Der Waals Corrections for the PtX_2 (X=O,S,Se) Compounds. *Physical Review B* **88,** 075421 (2013).
